# Systems analysis unravels a common rural-urban gradient in immunological profile, function, and metabolic dependencies

**DOI:** 10.1126/sciadv.adu0419

**Published:** 2025-04-30

**Authors:** Mikhael D. Manurung, Graham A. Heieis, Marion König, Shohreh Azimi, Malick Ndao, Tom Veldhuizen, Dennis Hoving, Pytsje T. Hoekstra, Yvonne C. M. Kruize, Linda J. Wammes, Roberta Menafra, Marouba Cisse, Souleymane Mboup, Alioune Dieye, Susan Kloet, Dicky L. Tahapary, Taniawati Supali, Manfred Wuhrer, Cornelis H. Hokke, Bart Everts, Ahmed Mahfouz, Simon P. Jochems, Maria Yazdanbakhsh, Moustapha Mbow

**Affiliations:** ^1^ Leiden University Center for Infectious Diseases (LU-CID), Leiden University Medical Center, Leiden, Netherlands.; ^2^ Department of Human Genetics, Leiden University Medical Center, Leiden, Netherlands.; ^3^ Department of Immunology, Faculty of Medicine, Pharmacy, and Odontology, Cheikh Anta Diop University of Dakar, Dakar, Senegal.; ^4^ Leiden Genome Technology Center, Leiden University Medical Center, Leiden, Netherlands.; ^5^ Institute of Health Research, Epidemiological Surveillance, and Training, Dakar, Senegal.; ^6^ Department of Internal Medicine, Faculty of Medicine, University of Indonesia, Jakarta, Indonesia.; ^7^ Department of Parasitology, Faculty of Medicine, University of Indonesia, Jakarta, Indonesia.; ^8^ Center for Proteomics and Metabolomics, Leiden University Medical Center, Leiden, Netherlands.

## Abstract

Urbanization affects environmental exposures and lifestyle, shaping immune system variation and influencing disease susceptibility and vaccine responses. Here, we present systems analysis of immune profiles across the rural-urban gradient, comparing rural and urban Senegalese with urban Dutch individuals. By integrating single-cell phenotyping, metabolic profiling, and functional analysis, we reveal a trajectory of immune remodeling along the gradient. This includes enrichment of proinflammatory CD11c^+^ B cells associated with altered IgG Fc glycosylation, adaptive NK cells with reduced responsiveness to accessory cytokines, and CD161^+^CD4^+^T cells with enhanced cytokine production in rural settings. Metabolic perturbation studies demonstrated distinct dependencies on glycolysis, pentose phosphate pathway, and fatty acid synthesis for cellular cytokine responses across populations. We validate core rural-urban immune signatures in an independent Indonesian cohort, suggesting shared immunological adaptations to urbanization across ancestries and geographical areas. Our findings provide insights into rural-urban immune function in understudied populations.

## Teaser

Integrative multi-omics analysis reveals distinct immune signatures along the rural-urban gradient across diverse populations.

## INTRODUCTION

Urbanization drives variations in immune profiles through differences in environmental exposure and lifestyle (*1*, *2*). These variations are evident not only between geographical locations (*3*) but also between rural and urban areas in the same location (*4*), with profound implications for both health and disease. For instance, influenza and tetanus vaccinations elicit lower immunogenicity in children from rural Gabon (*5*–*7*), a phenomenon known a vaccine hyporesponsiveness, which could lead to inadequate protection by several vaccines in rural settings (*8*). Conversely, the prevalence of immune-mediated diseases, such as inflammatory bowel diseases, is rising in both western and newly industrialized countries (*9*). These observations underscore the need for a deeper understanding of how environmental exposure and lifestyle affect immune variability to improve health interventions.

Recent studies have shed insights on key environmental drivers of immune variation (*2*, *10*, *11*). Cytomegalovirus (CMV) infection modulates immune function by expanding memory T cells and senescent cells, potentially compromising vaccine responses (*12*). Exposure to β-glucan, a fungal cell wall component, enhances monocyte responses to unrelated pathogens through “trained immunity” (*13*). Notably, most studies on human immune variability have focused on historically affluent countries, mirroring biases in genomic research (*14*), with few investigating immune profiles across the rural-to-urban gradient in low- and middle-income countries.

Emerging evidence suggests that distinct immune characteristics exist in rural African populations, with heightened activation, proinflammatory, and memory phenotypes, as well as altered immunoglobulin G (IgG) glycosylation profiles (*15*–*18*). These activated immune profiles, which may indicate higher exposures to microorganisms and parasites, are also reflected in the expansion of type 2 and regulatory immune cells in rural Indonesians, including interleukin-10–positive (IL-10^+^) B cells (*4*, *19*). Notably, the activated immune state of individuals living in rural areas could adversely affect vaccine responses, similar to what has been observed in the elderly (*20*). Furthermore, differences in dietary factors and metabolism have also been shown to modulate proinflammatory cytokine production in Tanzanian adults (*21*). These findings underscore the diversity of exposures and lifestyles across various population settings and their consequences on the immune system. To fully elucidate immune remodeling along the rural-urban gradient, an integrative multi-omics approach is needed to unravel the structure of the immune network regulating cell composition, functional responses, and cellular metabolism across populations.

Here, we conducted an integrative analysis of immune profiles in rural and urban Senegalese individuals and urban Dutch individuals using mass cytometry, spectral flow cytometry, cellular indexing of transcriptomes and epitopes (CITE-seq), and IgG Fc glycosylation profiling. Our data revealed a continuous trajectory of immune variation along the rural-urban gradient, characterized by distinct phenotypic, functional, and metabolic features. To consolidate our findings, we validated parts of the immune signature in an independent Indonesian cohort, indicating shared immunological adaptations to rural environments across diverse geographical locations. Last, we investigated shifts in cellular cytokine responses across populations under perturbation of key metabolic pathways.

## RESULTS

### Study population

**Fig. 1.**
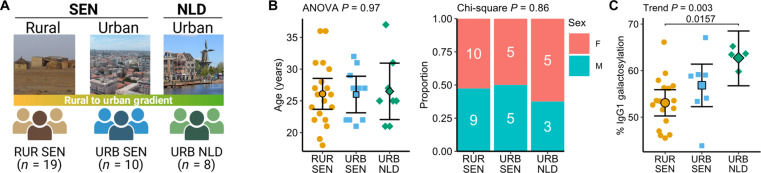
Study population characteristics. (**A**) Schematic representation of the study population. This figure was created using BioRender. Photographs representing RUR SEN, URB SEN, and URB NLD are credited to: WILLAV-FR, Initsogan, and W. Bulach, respectively (Wikimedia Commons). (**B**) Age distribution (dot plot) and sex distribution (stacked bar plot) across residential areas. Points represent individual samples; error bars show adjusted marginal means with 95% confidence intervals (CIs). *P* values comparing differences in age and sex distributions are indicated. (**C**) IgG1 Fc galactosylation levels across residential areas. Points represent individual samples; error bars show adjusted marginal means with 95% CIs. Trend test and Tukey’s-corrected pairwise comparisons *P* values are indicated. RUR, rural; URB, urban; SEN, Senegal; NLD, the Netherlands; F, female; M, male. ANOVA, analysis of variance.

To characterize immune profiles along the rural-to-urban gradient, we analyzed 37 age- and sex-matched healthy adults from rural and urban Senegal (Western Africa) and urban Netherlands (Western Europe) (Fig. 1A). The median age of all participants was 26 years (range, 18 to 37 years), with 57% female representation; there were no significant differences in age and sex across groups (Fig. 1B and table S1). All Senegalese participants had resided in their respective areas for at least 10 years (table S2). Rural Senegalese exhibited characteristics typical of a rural lifestyle, such as larger household sizes, increased animal contact, and higher occurrence of earth floors and mud walls in their residences (table S2).

Because IgG1 Fc galactosylation may characterize populations from different environmental settings, with low galactosylation pointing toward more rural residence (*18*), we analyzed the levels of this marker in our study cohort to validate the rural/urban characteristics concerning the intensity of exposure to microorganisms and parasites. We observed that IgG1 Fc galactosylation levels were the lowest in rural Senegalese, followed by urban Senegalese and the Dutch (trend test *P* = 0.003; Fig. 1C). Peripheral blood eosinophil frequencies were fivefold higher in rural Senegal dwellers compared to urban Senegal and Netherlands (mean 10.9% versus 2.1% versus 1.8%, respectively; trend test *P* = 0.002; fig. S1A), which could be attributed to either past or present helminth infections.

Considering the high prevalence of schistosomiasis in our rural study sites, we also performed the highly sensitive circulating anodic antigen (CAA) assay to supplement microscopy results. The CAA assay was positive in 47% (table S1) of rural Senegalese participants, indicating low-intensity schistosomiasis, while all urban Senegalese and Dutch participants tested negative. IgG1 Fc galactosylation levels did not differ significantly between CAA^+^ and CAA^−^ rural participants (*P* = 0.21; fig. S1B), suggesting that low-intensity schistosomiasis did not substantially influence this marker. In addition, the analysis of circulating inflammatory markers revealed distinct patterns across populations: Rural Senegalese exhibited elevated C5a and IL-8 [trend test false discovery rate (FDR) = 0.045 and 0.064, respectively], whereas urban Dutch individuals showed higher CCL2 levels (*P* = 0.004 versus rural Senegalese; fig. S1C and table S3). Together with socioeconomic indicators, these immune biomarker profiles supported our rural/urban classification of the study participants.

### Integrative analysis reveals immune variation trajectory along the rural-urban gradient

**Fig. 2.**
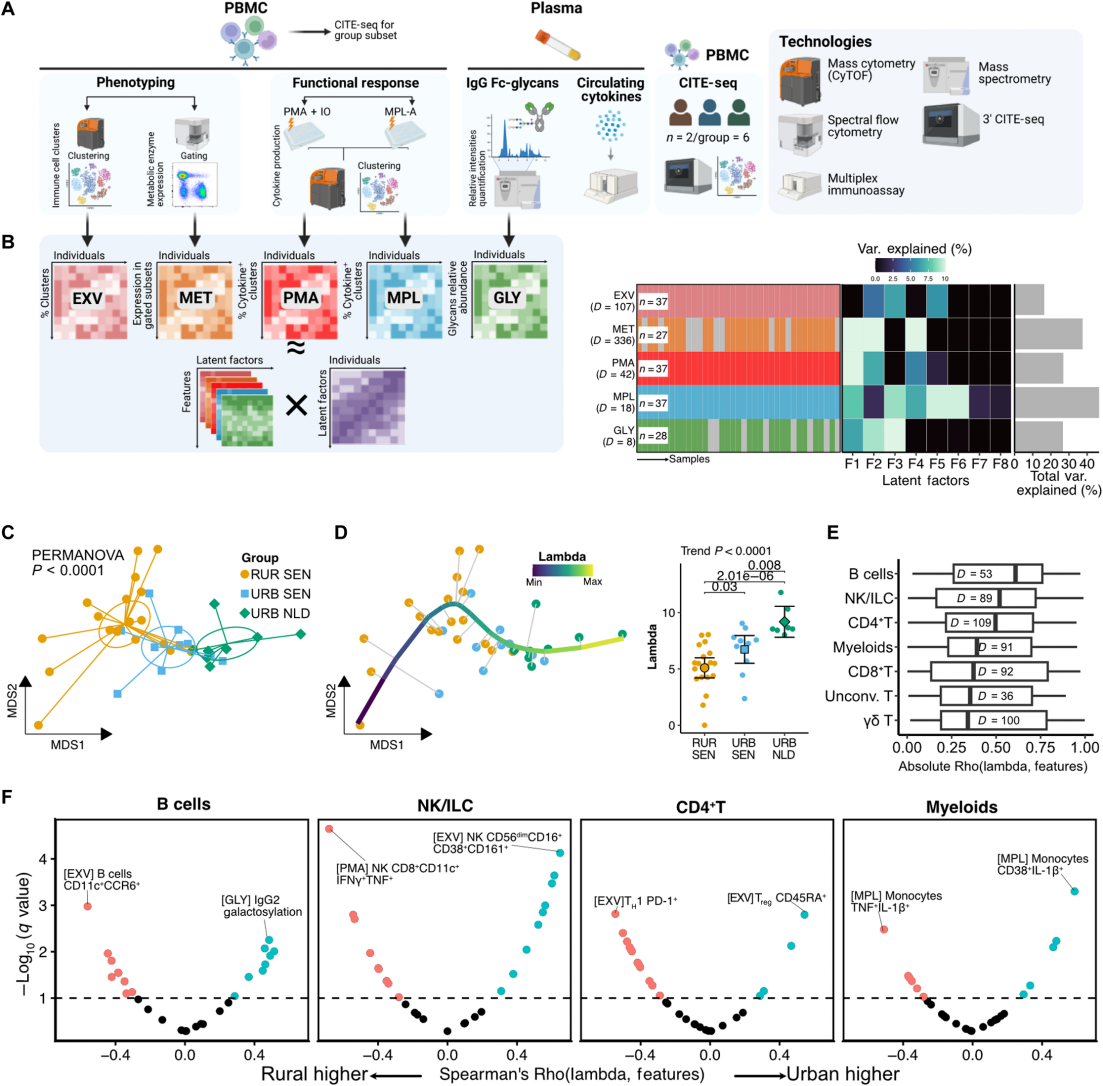
Integrative analysis revealed a rural-to-urban immune trajectory. (**A**) Overview of samples and assays performed. (**B**) MOFA of five datasets showing variance explained per dataset (bar plot) and latent factor (center heatmap), with sample numbers (*n*) and feature dimensionalities (*D*). Panels (A) and (B) were created using BioRender. (**C**) MDS plot using MOFA factors. Points represent individual samples colored by residential group. *P* values indicate group centroid comparisons using PERMANOVA test. (**D**) Principal curve trajectory analysis showing sample projections (left) and lambda parameter distribution across residence groups (right). Error bars represent adjusted marginal means with 95% CIs. Linear trend test and Conover post hoc test for pairwise comparisons *P* values are indicated. (**E**) Spearman’s correlation distribution between lambda and immune features grouped by immune cell lineage, sorted by median absolute correlation. Number of features associated with each lineage (D) is shown. (**F**) Volcano plots of immune features correlated with rural-to-urban trajectory (lambda) for the top four immune lineages. PMA; EXV, ex vivo PBMC phenotyping dataset; MET, ex vivo PBMC metabolic enzyme dataset; PMA, PMA/ionomycin-stimulated PBMC dataset; MPL, MPL-stimulated PBMC dataset; GLY, IgG Fc glycosylation dataset; Unconv. T, unconventional T cells.

To characterize immune profiles across the rural-urban gradient, we acquired six datasets encompassing peripheral blood immune cell phenotyping [ex vivo phenotyping (EXV) and metabolic enzyme expression (MET) datasets], cellular cytokine production analysis following stimulations [phorbol 12-myristate 13-acetate (PMA)/ionomycin and monophosphoryl lipid-A (MPL) datasets], IgG Fc glycan profiling [IgG glycosylation (GLY) dataset], and, from a subset of individuals, CITE-seq (Fig. 2A and table S4).

We applied multi-omics factor analysis (MOFA) to the EXV, MET, PMA, MPL, and GLY datasets (Fig. 2B). MOFA learns latent factors capturing principal sources of variation across datasets in an unsupervised manner (*22*). Among the eight latent factors learned by MOFA, factors one (trend test *P* = 0.003), two (*P* < 0.0001), and three (*P* < 0.0001) were significantly associated with the rural-to-urban gradient (fig. S2A). Multidimensional scaling (MDS) analysis using all eight latent factors as input revealed significant separation of groups, not only between the Senegalese and Dutch individuals but also between rural and urban Senegalese [permutational multivariate analysis of variance (PERMANOVA) *P* < 0.0001; Fig. 2C]. Moreover, MDS analysis using MOFA factors resulted in the highest variance explained by residence [PERMANOVA coefficient of determination (*R*^2^) = 0.4; Fig. 2C] compared to any single omics analysis (highest PERMANOVA *R*^2^ = 0.142, MPL dataset; fig. S2B). These results demonstrate that integrative multi-omics analysis using MOFA captured relevant rural-to-urban immune variation more effectively than single omics analysis.

To investigate whether immune variation across the study participants lies along a continuum (*23*), we used principal curve analysis. This unsupervised approach identified a trajectory spanning from rural Senegalese to urban Dutch individuals (Fig. 2D). Analysis of the lambda metric, which quantifies a sample’s position along the principal curve, revealed that individuals were ordered according to our predefined rural-urban gradient (trend test *P* < 0.0001), with significant stepwise differences between rural and urban Senegalese (*P* = 0.03) as well as between urban Senegalese and urban Dutch participants (*P* = 0.008; Fig. 2D). Given this validation of the rural-urban ordering, we used it as the basis for subsequent statistical analyses. Correlation analysis between the lambda metric and features from all five datasets (Fig. 2E) revealed that the rural immune trajectory was characterized by proinflammatory profiles, which include higher frequencies of CD11c^+^ B cells, interferon-γ–positive (IFN-γ^+^)tumor necrosis factor–positive (TNF^+^) natural killer (NK) cells, programmed cell death 1–positive (PD-1^+^) T helper 1 (T_H_1) cells, and TNF^+^IL-1β^+^ monocytes (Fig. 2F). In summary, unsupervised integrative analysis using MOFA revealed a rural-to-urban trajectory of immune variation, with both innate and adaptive immune features driving this variation.

### Higher frequencies of proinflammatory CD11c^+^ B cells in rural settings were associated with altered IgG Fc glycosylation patterns

**Fig. 3.**
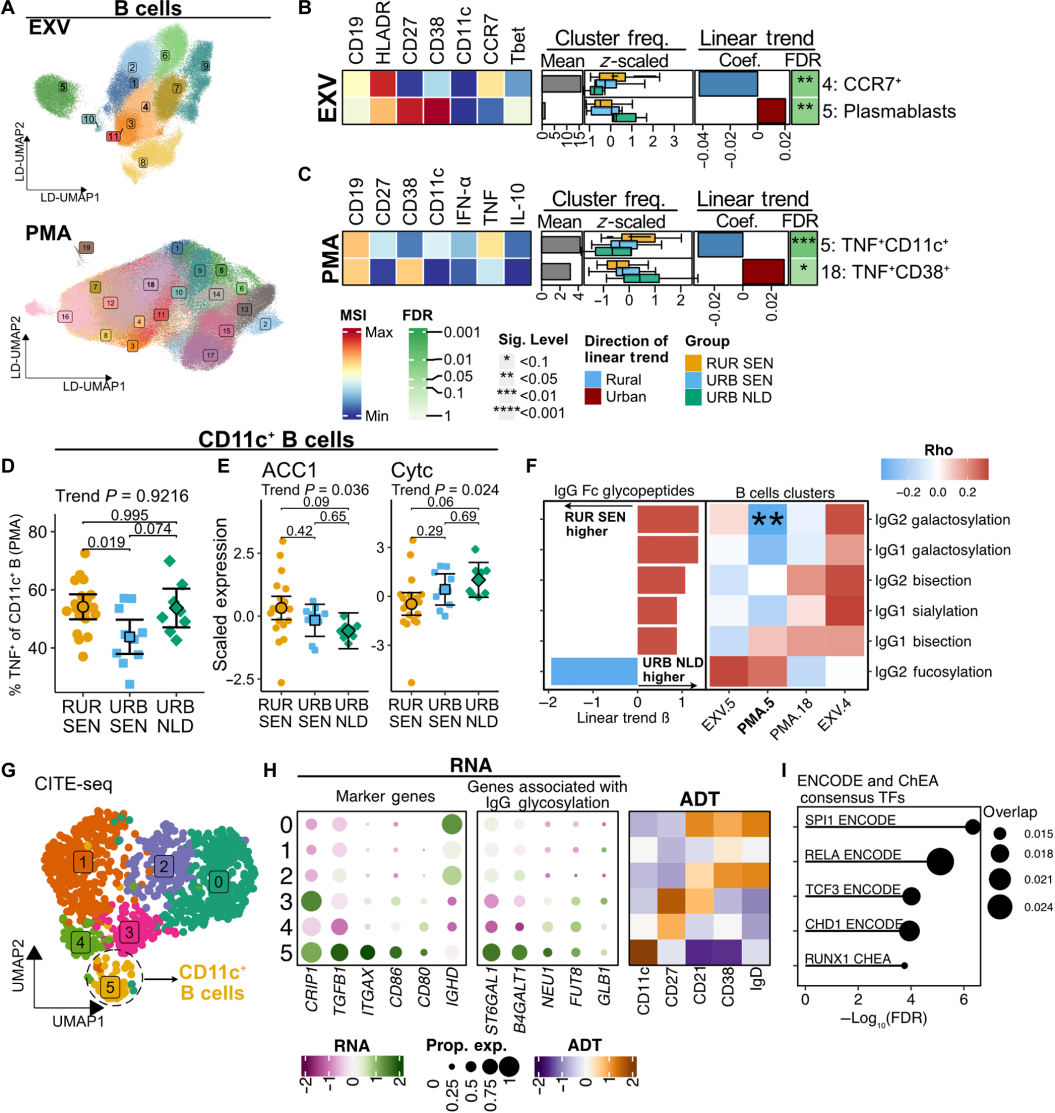
TNF^+^CD11c^+^ B cell frequencies are associated with altered IgG glycosylation profiles. (**A**) LD-UMAP plots of B cells subsampled from EXV and PMA datasets with cluster annotations. (**B** and **C**) Heatmaps of differentially abundant (trend test FDR < 0.1) clusters in EXV (B) and MPL (C) datasets showing median signal intensity (tile colors), mean cluster frequencies relative to lineage (gray bars), *z*-scored cluster frequencies by residence groups (boxplots), as well as trend test coefficients (red/blue bars) and FDR (asterisks). (**D** and **E**) TNF-producing cell frequencies (D) and metabolic enzyme levels (E) in CD11c^+^ B cells across populations. Points represent individual samples; error bars show adjusted marginal means with 95% CIs. Trend test and Tukey’s-corrected pairwise *P* values are indicated. (**F**) Left: Linear trend effect sizes of differentially abundant IgG Fc glycopeptides (FDR < 0.1). Right: Spearman’s correlations between IgG Fc-glycan abundances and CD11c^+^ B cell cluster frequencies. Asterisks indicate correlation *P* value. (**G**) UMAP plot of B cells from CITE-Seq data with CD11c^+^ B cells encircled (dashed lines). (**H**) Heatmaps showing *z*-scored expression of cluster markers and IgG glycosylation pathway genes (RNA) and proteins (ADT). (**I**) Top 5 enriched transcription factors in CD11c^+^ B cells. Dot size indicates gene list overlap. Enrichment analysis was performed using the Enrichr web interface (accessed August 2023) using ENCODE and ChEA consensus TF gene sets. MSI, median signal intensity; ADT, antibody-derived tags; UMAP, uniform manifold approximation; LD-UMAP, UMAP with linear discriminant initialization; *P/FDR < 0.1, **P/FDR < 0.05, ***P/FDR < 0.01, and ****P/FDR < 0.001.

Given the strong correlation of B cell features with the rural-to-urban trajectory, we performed a detailed differential abundance (DA) analysis on B cell subsets and clusters identified by mass cytometry (Fig. 3A). At the subset level, we found higher frequencies of CD11c^+^ B cells in Senegalese individuals than in Dutch individuals (fig. S3A). Cluster-level analysis additionally revealed the enrichment of CCR7^+^ B cells (cluster 4) in rural settings and CD27^+^CD38^hi^ plasmablasts (cluster 5) in Dutch individuals (Fig. 3B, fig. S3B, and table S5).

Next, we assessed the cytokine response of B cells following PMA (Fig. 3A, fig. S3C, and table S5). The frequencies of TNF-producing CD11c^+^ B cells (cluster 5) were highest in rural Senegalese, followed by urban Senegalese and Dutch individuals (Fig. 3C), with higher frequencies of TNF^+^ cells in the CD11c^+^ B cells of rural Senegalese (Fig. 3D). These results, together with the CD11c^+^ B cell frequency data, indicate that rural-to-urban immune variation within the B cell compartment is characterized by the enrichment of proinflammatory CD11c^+^ B cells in rural settings.

Metabolic enzyme analysis in CD11c^+^ B cells suggests an association between proinflammatory cytokine production and metabolic rewiring. Trend analysis showed higher acetyl-CoA carboxylase (ACC1) and lower cytochrome *c* (Cytc) levels in rural individuals (Fig. 3E), suggesting a shift from oxidative phosphorylation to anabolic metabolism with increased fatty acid synthesis (FAS) to support their proinflammatory profile. To determine whether low-intensity schistosomiasis influenced the above signatures, we compared B cell features between CAA-positive and CAA-negative rural Senegal dwellers, which revealed no significant differences in any of these features (fig. S4, and table S6).

Given the crucial role of B cells in modulating IgG glycosylation in response to environmental stimuli (*24*, *25*) and the potential of CD11c^+^ B cells to differentiate into antibody-secreting cells (*26*, *27*), we investigated IgG Fc-glycosylation patterns across the rural-to-urban gradient and their association with proinflammatory CD11c^+^ B cells. In addition to the higher IgG1 Fc galactosylation levels (Fig. 1C) in rural Senegalese, we observed variations in other IgG Fc glycopeptides. Fc fucosylation levels of IgG1 and IgG2 were higher in rural settings, whereas Fc galactosylation of IgG1 and IgG2 was higher in urban settings (Fig. 3F). Notably, IgG2 galactosylation and IgG3/4 sialylation levels were significantly correlated with the frequency of differentially abundant TNF^+^CD11c^+^ B cells (cluster PMA.5; Fig. 3F).

We next analyzed the B cells CITE-seq data (Fig. 3G) to provide insights into the functions of CD11c^+^ B cell (cluster 5), which was characterized by low levels of CD21 and high *CD80*, *CD86*, and *TGFB1* (Fig. 3H). Focusing on genes related to IgG Fc glycosylation pathways, we found that CD11c^+^ B cells highly expressed *ST6GAL1*, *B4GALT1*, *NEU1*, *FUT8*, and *GLB1* (Fig. 3H) (*25*, *28*, *29*). Compared to memory B cells (clusters 3 and 4), CD11c^+^ B cells showed increased expression of interferon signaling genes (*IFITM2* and *IFI30)*, *TGFB1*, and *HLA-DRB1.* Transcription factor enrichment analysis suggested that RelA (p65), a component of the nuclear factor κB (NF-κB) pathway (*30*), might regulate CD11c^+^ B cell transcriptome (Fig. 3I). Together, these data suggest that CD11c^+^ B cells have a distinct signature from memory B cells, which might drive their inflammatory profile seen in rural participants.

In conclusion, rural-to-urban variation within the B cell compartment is characterized by the enrichment of CD11c^+^ cells expressing proinflammatory cytokines in rural settings. These CD11c^+^ B cells, which exhibit metabolic rewiring toward increased FAS, are also associated with altered IgG glycosylation profiles.

### Enrichment of adaptive NK cells correlates with attenuated response to MPL in rural settings

**Fig. 4.**
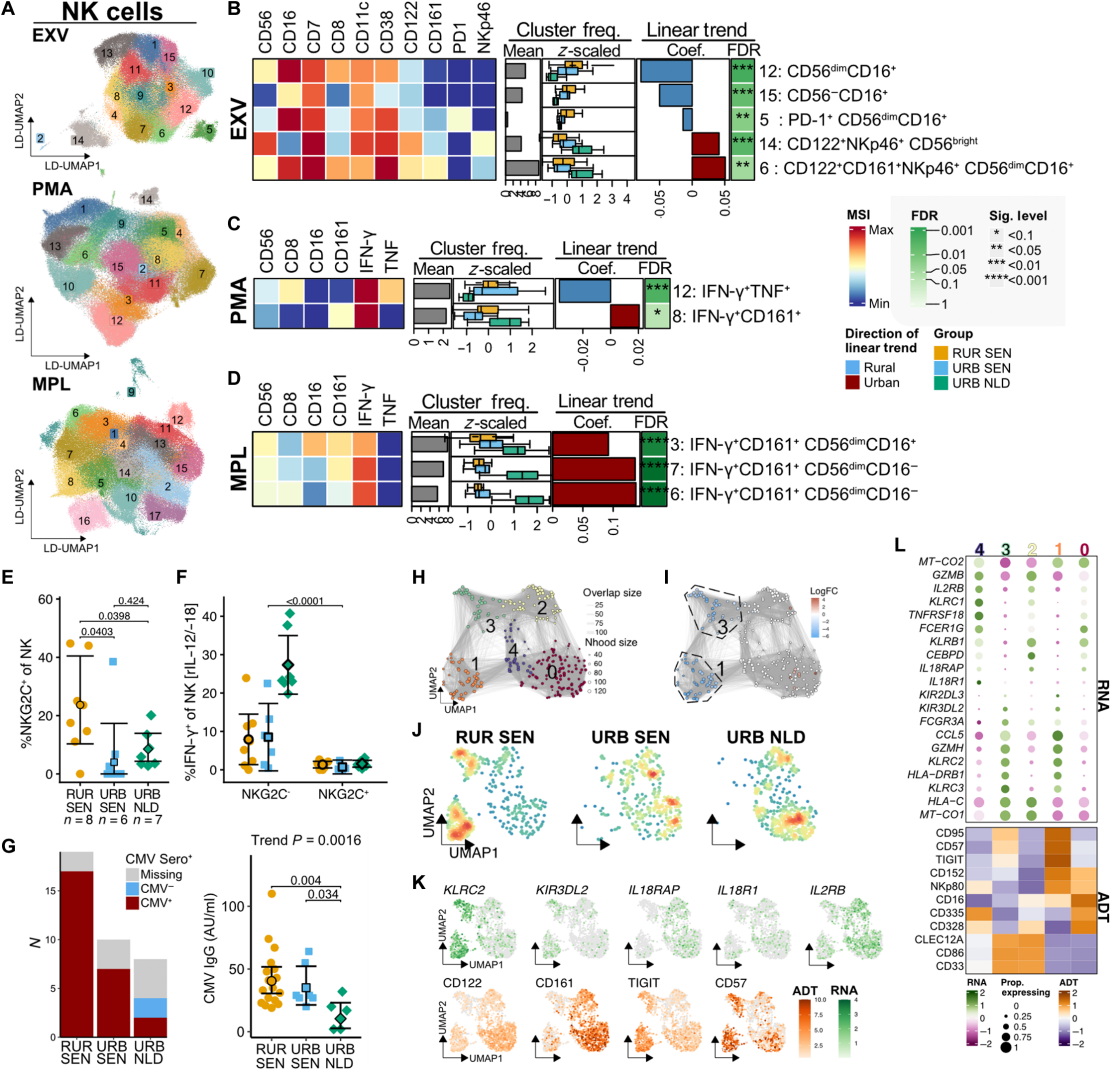
Lower frequencies of IFN-γ^+^ NK cells in rural Senegalese after MPL stimulation are associated with the enrichment of adaptive NK cells. (**A**) LD-UMAP of NK cells subsampled from EXV, PMA, and MPL datasets with cluster annotations. Colors and labels indicate cell clusters. (**B** to **D**) Heatmaps of differentially abundant (trend test FDR < 0.1) clusters in EXV (B), PMA (C), and MPL (D) datasets showing median signal intensity (tile colors), mean cluster frequencies relative to lineage (gray bars), *z*-scored cluster frequencies by residence groups (boxplots), as well as trend test coefficients (red/blue bars) and FDR (asterisks). (**E**) NKG2C^+^ NK cell frequencies from an independent cohort (*n* = 8, 6, and and 7 for RUR SEN, URB SEN, and URB NLD, respectively); pairwise comparisons *P* values are shown. (**F**) IFN-γ^+^ NK cell frequencies after IL-12/IL-18 stimulation in NKG2C^+^ versus NKG2C^−^ NK cells. (**G**) CMV serostatus (left) and anti-CMV IgG titers (right) across populations. Points represent individuals and error bars adjusted marginal means with 95% CIs. Trend test and Tukey’s-corrected *P* values are indicated. (**H** and **I**) CITE-seq neighborhood graphs showing cluster assignments (H) and differentially abundant neighborhoods at 10% FDR (I). (**J** and **K**) UMAP of NK cells from CITE-seq data showing cell density (J) and RNA/ADT expression (K). (**L**) Heatmaps of marker genes (RNA) and proteins (ADT) in NK cell clusters. Colors indicate expression *z* scores; circle size indicates proportion of cells expressing each gene. CMV, cytomegalovirus. *P/FDR < 0.1, **P/FDR < 0.05, ***P/FDR < 0.01, and ****P/FDR < 0.001. FC, fold change.

The second cell lineage that was highly correlated with the rural-to-urban gradient was NK cells and innate lymphocytes (ILCs). To characterize variations in the composition of these cells along the gradient, we performed DA analysis on the EXV dataset (Fig. 4A). Subset-level analysis revealed enrichment of CD56^−^CD16^+^ and CD56^dim^CD16^−^ NK subsets in rural settings, while CD56^bright^ NK (immature NK) and ILC3 subsets were enriched in the Dutch volunteers (fig. S5A). Cluster-level analysis showed that clusters enriched in rural individuals (clusters 5, 12, and 15) exhibited lower expression of CD122 (IL-2/IL-15 receptor subunit β), CD161, and NKp46 compared to clusters enriched in urban individuals (clusters 6 and 14; Fig. 4B, fig. S5B, and table S7). Notably, a PD-1–expressing NK cluster was enriched in rural settings (cluster 5; Fig. 4B). These findings indicate that the NK compartment in rural settings is characterized by mature (CD56^−/dim^) NK cells with low CD122 and CD161 expression.

When examining these cells functionally, we observed comparable IFN-γ^+^ or TNF^+^ cell frequencies following PMA stimulation at the subset level (fig. S5E). However, cluster-level analysis showed higher frequencies of IFN-γ^+^TNF^+^ NK (cluster 12) in rural settings and IFN-γ^+^CD161^+^ NK (cluster 8) in urban settings (Fig. 4C, fig. S5C, and table S7). Regarding response following MPL stimulation, we observed higher frequencies of IFN-γ^+^CD161^+^ NK cell clusters in urban settings (Fig. 4D, fig. S5D, and table S7), with a higher proportion of IFN-γ^+^ cells within the CD161^+^ NK cell population (trend test *P* < 0.0001; fig. S5F). Differential analysis comparing NK cell parameters between rural Senegal dwellers with (CAA^+^) and without low-intensity schistosomiasis (CAA^−^) showed no significant differences in any features across all datasets (fig. S4 and table S6).

Given that NK cells generally lack TLR4 (*31*, *32*), their IFN-γ production in response to MPL is likely mediated indirectly through accessory cytokines (IL-12/-15/-18) (*33*), which may be produced by activated myeloid cells. Our previous observation of lower CD122^+^ (IL-15 receptor subunit) NK cell cluster frequencies in rural settings suggests a potential mechanism for their attenuated MPL responsiveness, possibly due to decreased sensitivity to these accessory cytokines. This functional characteristic is exhibited by a subset of NK cells expressing NKG2C (*33*, *34*). In an additional set of rural-urban Senegalese individuals, we found higher frequencies of NKG2C^+^ cells in the NK cell compartment of rural compared to both urban Senegalese (*P* = 0.0403) and urban Dutch individuals (*P* = 0.0398) (Fig. 4E). Upon stimulation with IL-12 and IL-18, NKG2C^+^ NK cells exhibited a lack of IFN-γ response compared to their NKG2C^−^ counterpart (*P* < 0.0001; Fig. 4F). The expansion of NKG2C^+^ NK cells, also known as adaptive NK cells, could be driven by CMV infection (*34*, *35*). However, considering that both rural and urban Senegalese individuals are all CMV seropositive and showed comparable IgG titers (Fig. 4G), additional factors might also affect the frequencies of this immune subset.

CITE-seq analysis corroborated these findings, revealing an enrichment of adaptive NK cell clusters 1 and 3 in rural Senegalese (Fig. 4, H to J). These clusters coexpressed *KLRC2*/NKG2C, CD57, and checkpoint receptors including CTLA-4 and TIGIT (Fig. 4, K and L). Furthermore, these clusters exhibited low expression of genes encoding accessory cytokine receptors (*IL2RB*/*IL15RB*, *IL18R1*, and *IL18RAP*) as well as CD122 and CD161 (Fig. 4, K and L), resembling the phenotype of NK cell clusters 5, 12, and 15 identified in the EXV dataset.

In summary, NK cells in rural settings showed a more differentiated phenotype with increased expression of checkpoint markers, attenuated response to accessory cytokines, and reduced expression of cytokine receptors. These phenotypic alterations were associated with differential responsiveness to MPL adjuvant stimulation.

### Higher proinflammatory cytokine response by CD161^+^CD4^+^T cells in rural settings is associated with heightened basal expression of IFN-signaling genes

**Fig. 5.**
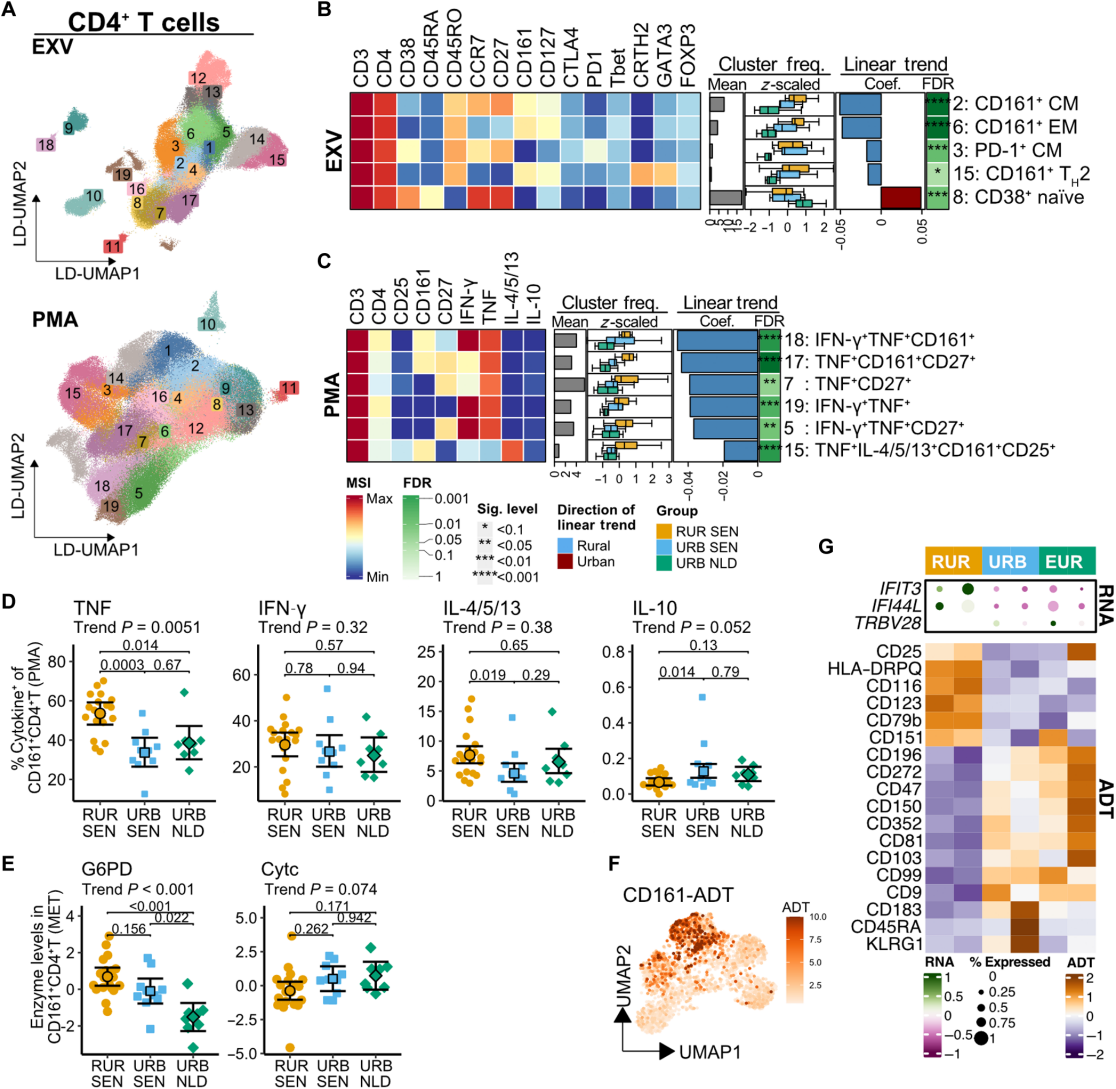
CD161^+^CD4^+^T cells are enriched in rural Senegalese, produce more proinflammatory cytokines, and express more IFN-related genes in the resting state. (**A**) LD-UMAP of CD4^+^T cells subsampled from EXV and MPL datasets with cluster labels. (**B** and **C**) Heatmaps of differentially abundant (trend test FDR < 0.1) CD4^+^T cell clusters in EXV (B) and PMA (C) datasets showing median signal intensity (tile colors), mean cluster frequencies relative to lineage (gray bars), *z*-scored cluster frequencies by residence groups (boxplots), as well as trend test coefficients (red/blue bars) and FDR (asterisks). (**D**) Cytokine-producing CD161^+^CD4^+^T cell frequencies across residence groups with 95% CIs, trend test, and Tukey’s-corrected *P* values. (**E**) Metabolic enzyme levels in CD161^+^CD4^+^T cells with 95% CIs and trend test *P* values. (**F**) UMAP of CD4^+^T cells from CITE-seq data showing CD161 expression. (**G**) Differentially expressed genes/proteins in CD161^+^CD4^+^T cells comparing rural Senegalese to urban Senegalese/Dutch individuals. Colors indicate expression *z* scores; circle size indicates proportion of cells expressing each gene. *P/FDR < 0.1, **P/FDR < 0.05, ***P/FDR < 0.01, and ****P/FDR < 0.001.

CD4^+^T cells emerged as the third-ranked immune cell lineage correlated with the rural-to-urban gradient. To identify CD4^+^T cell subsets driving this gradient, we performed DA analysis on subsets and clusters from the EXV mass cytometry data (Fig. 5A). At the subset level, central and effector memory subsets as well as T_H_2 cells, were enriched in rural settings, while naïve T cells and regulatory T (T_reg_) cells were enriched in urban settings (fig. S6A). Cluster-level analysis revealed higher frequencies of CD4^+^T cell clusters expressing CD161 (clusters 2, 6, and 15) or CTLA-4^+^PD-1^+^ (cluster 3) in rural Senegalese individuals, followed by urban Senegalese and Dutch individuals (Fig. 5B, fig. S6B, and table S8). Notably, cluster 15 exhibited a CD161^+^CRTH2^+^CD27^low^ phenotype, consistent with the pathogenic effector T_H_2A subset (*36*). Conversely, Dutch individuals showed higher frequencies of naïve CD4^+^T cells expressing CD38 (cluster 8; Fig. 5B), associated with lower metabolic activity (*37*).

Upon PMA stimulation, higher frequencies of seven CD4^+^T clusters producing T_H_1 or T_H_2 cytokines in rural Senegalese were seen (Fig. 5C, fig. S6C, and table S8), three of which expressed CD161 (clusters 15, 17, and 18). Further analysis revealed that the frequency of TNF-producing CD161^+^CD4^+^T cells was higher in rural Senegalese compared to urban Senegalese or Dutch individuals (Fig. 5D). These results indicate that the variation in CD4^+^T cell compartment was driven by the enrichment of proinflammatory subsets in rural settings, with enriched cytokine production in CD161-expressing cells.

Metabolic enzyme analysis of these CD161^+^CD4^+^T cells revealed higher glucose-6-phosphate dehydrogenase (G6PD) levels (trend test *P* < 0.001) and trends toward lower Cytc (*P* = 0.074) in rural Senegalese, followed by urban Senegalese and Dutch individuals (Fig. 5E), suggesting metabolic rewiring of this subset. Differential analysis comparing CD4^+^T cell features between rural participants with and without low-intensity schistosomiasis showed no significant differences across all datasets (fig. S4 and table S6). The CITE-seq analysis of CD161^+^CD4^+^T cells (Fig. 5F) revealed higher levels of IFN-related genes (IFIT3 and IFI44L) and CD151, a T cell activation marker (*38*), and lower levels of the inhibitory molecule BTLA (B- and T-lymphocyte attenuator, CD352) in rural Senegalese (Fig. 5G).

In summary, the higher frequencies of cytokine-producing CD4^+^T cells in rural Senegalese were attributed to enrichment of proinflammatory CD161^+^CD4^+^T cells with increased G6PD.

### Increased proinflammatory activity of monocytes in rural settings upon activation and at basal state

**Fig. 6.**
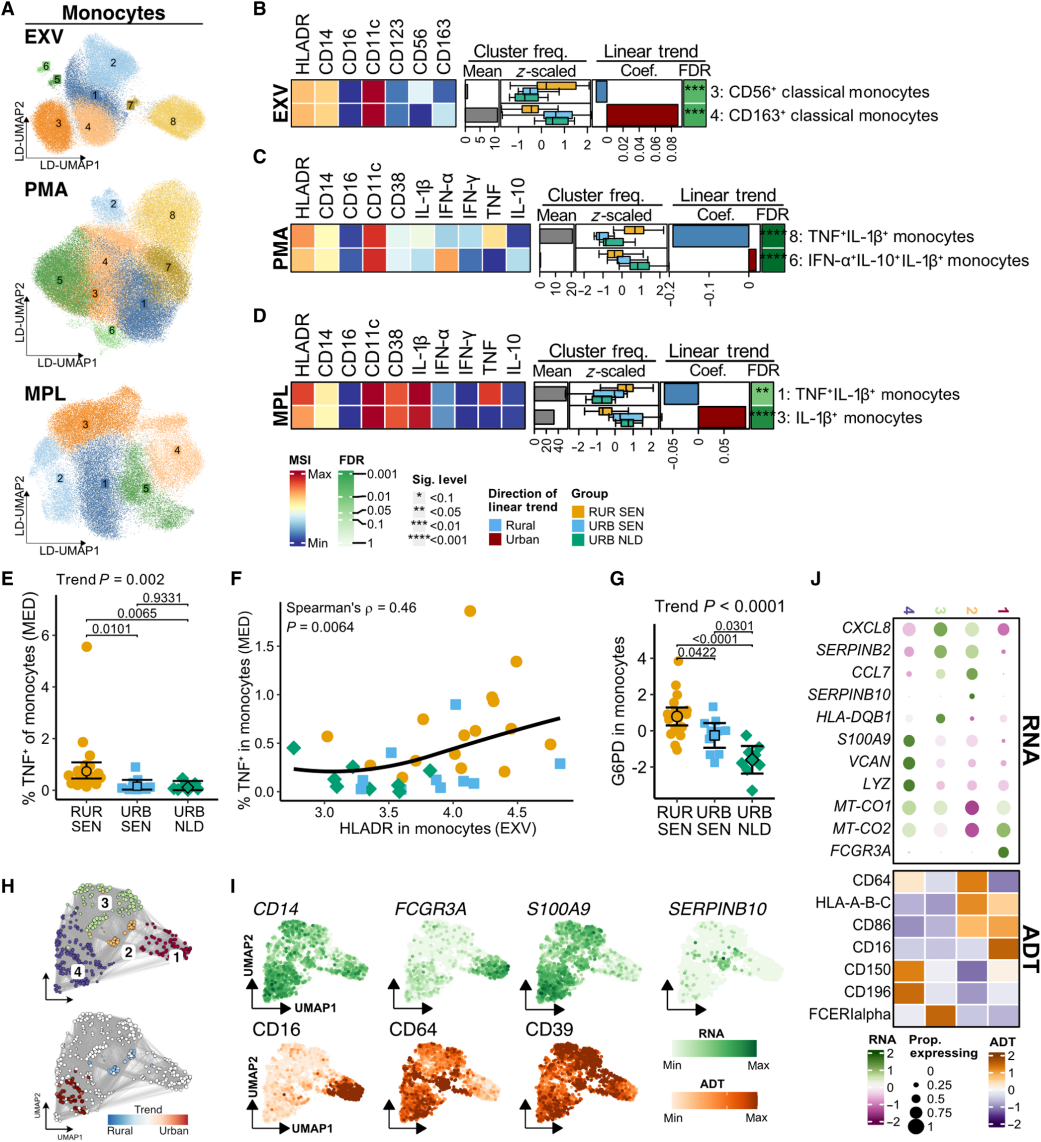
Higher basal activation of monocytes in rural Senegalese cells produces more proinflammatory cytokines following activation. (**A**) LD-UMAP of monocytes subsampled from EXV, PMA, and MPL datasets with cluster labels. (**B** to **D**) Heatmaps of differentially abundant (trend test FDR < 0.1) monocyte clusters in EXV (B), PMA (C), and MPL (D) datasets showing median signal intensity (tile colors), mean cluster frequencies relative to lineage (gray bars), *z*-scored cluster frequencies by residence groups (boxplots), as well as trend test coefficients (red/blue bars) and FDR (asterisks). (**E**) TNF^+^ monocyte frequencies after 6 hours of culture in medium. Points represent individuals, and error bars adjusted means with 95% CIs; trend test and Tukey’s-corrected *P* values are shown. (**F**) Correlation between HLA-DR expression (EXV) and spontaneous TNF production. (**G**) G6PD levels in monocytes across residence groups. (**H**) Neighborhood graphs showing cluster assignments (top) and differentially abundant neighborhoods (FDR < 10%; bottom). (**I**) UMAP of CITE-seq data showing RNA (top) ADT (bottom) expressions. (**J**) Heatmaps of monocyte cluster marker genes (RNA) and proteins (ADT). Colors indicate expression *z* scores; circle size indicates proportion of cells expressing each gene. *P/FDR < 0.1, **P/FDR < 0.05, ***P/FDR < 0.01, and ****P/FDR < 0.001.

Monocytes, which correlated with the rural-urban immune trajectory (Fig. 6A), when analyzed, showed no significant differences in cell abundances among groups (fig. S7A), but at the cluster level, higher frequencies of CD56^+^ and lower CD163^+^ classical monocytes were seen in rural Senegalese (Fig. 6B, fig. S7B, and table S9). CD56^+^ monocytes have been previously linked to inflammatory responses in rheumatoid arthritis (*39*) and as a prognostic marker in patients with coronavirus disease 2019 (COVID-19) (*40*, *41*).

When investigating these cells functionally, we found a heightened proinflammatory response in monocytes from rural individuals, both at basal state and upon activation. Higher frequencies of TNF^+^ monocyte clusters were found in rural settings upon stimulation with either PMA (Fig. 6C, fig. S7C, and table S9) or MPL (Fig. 6D, fig. S7D, and table S9). Monocytes of these rural individuals also showed the highest frequencies of spontaneous TNF production, followed by urban Senegalese and Dutch individuals (trend test *P* = 0.002; Fig. 6E). This profile correlated positively with mean monocytic HLA-DR levels (Spearman’s ρ = 0.46, *P* = 0.0065; Fig. 6F). Metabolic enzyme analysis showed higher G6PD levels in rural monocytes (Fig. 6G), suggesting that the proinflammatory profile might be associated with rewiring toward the pentose phosphate pathway (PPP). Differential analysis comparing monocytes parameters between rural participants with and without low-intensity schistosomiasis showed no significant differences across all datasets (fig. S4 and table S6).

CITE-seq further elucidated the proinflammatory profile of monocytes in rural individuals. We found that neighborhoods of cluster 2 were enriched in rural settings and cluster 4 in urban (Fig. 6H). Cluster 2 highly expressed the CD86 costimulatory molecule (*42*), CD64/FcγRI (*43*), and *HLA-ABC* (Fig. 6, I to J), suggesting that the activated monocytes in rural individuals were primed for antigen presentation (*44*). Moreover, cluster 2 also expressed *SERPINB10*, suggesting a compensatory mechanism to withstand inflammation-induced cell death (*45*).

### Rural-to-urban immune signatures identified in the Senegalese cohort can be validated in a distinct geographical location and ancestry

After characterizing signatures of immune variation along the rural-to-urban gradient in Senegalese and Dutch individuals, we investigated whether these signatures could be found in other populations. To this end, we selected subsets with strong discriminative power and examined their frequencies in an independent cohort.

**Fig. 7.**
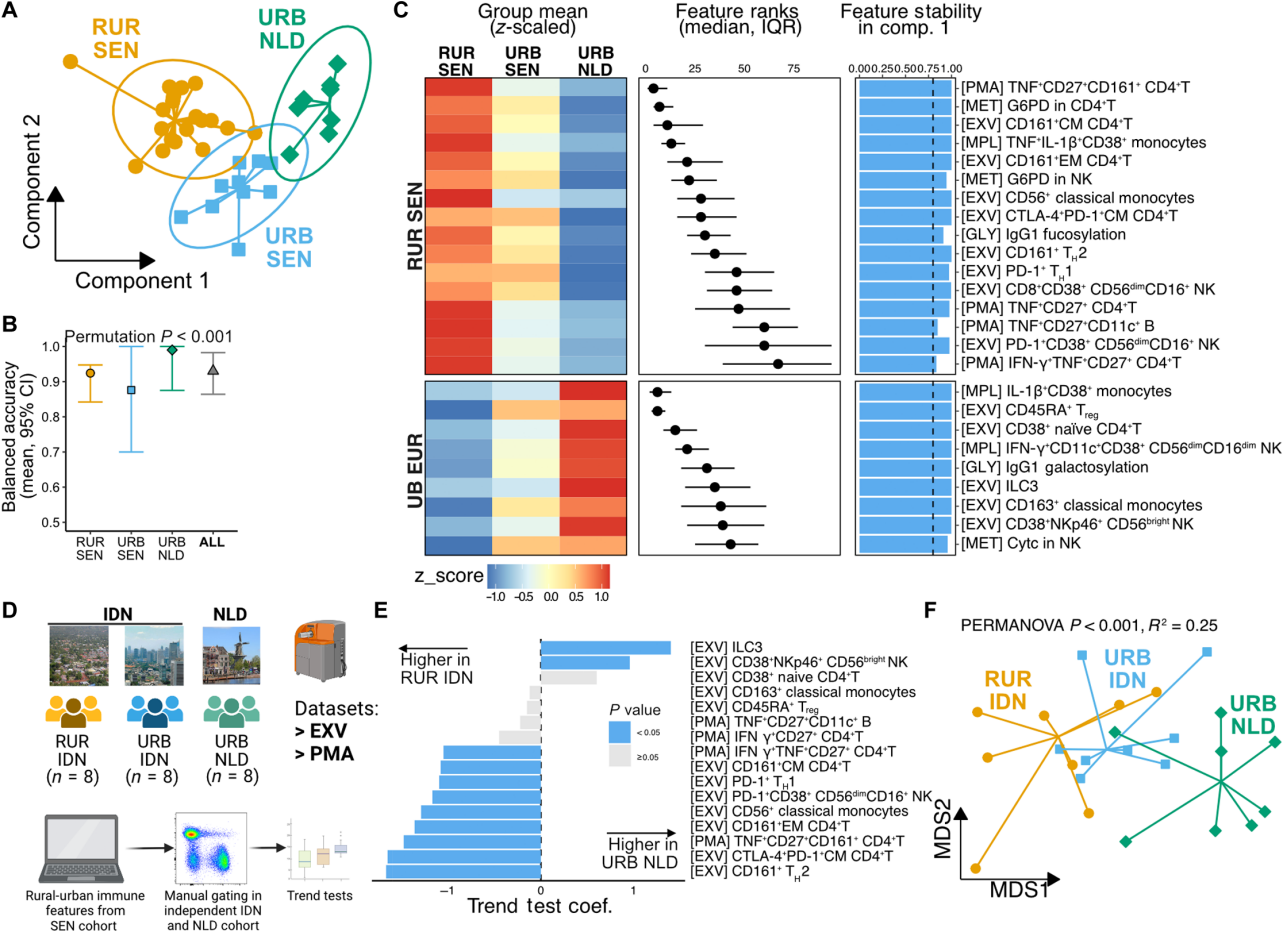
Validating rural-urban immune signatures in an independent, geographically and ancestrally distinct cohort. (**A**) Consensus ordination of samples obtained showing separation by residence group with 95% CIs. (**B**) Classification accuracies of the DIABLO model (mean and 95% CI) from 100 repetitions of fivefold cross-validation, with permutation test *P* value (DIABLO.test function in RVAideMemoire R package). (**C**) Stable features (>80%) distinguishing rural Senegalese (top) from urban Dutch (bottom) showing *z*-scaled mean values (heatmap tile colors), feature importance ranks (pointrange; calculated using OmicsFold R package), and selection frequencies across cross-validation folds (blue bars). (**D**) Schematic showing approach to validate signatures shown in C in an independent Indonesian-Dutch cohort. This figure was created using BioRender. Photographs representing RUR IDN, URB IDN, and URB NLD are credited to: Junior Jumper, Kevin Aurell, and W. Bulach, respectively (Wikimedia Commons). (**E**) Trend test coefficients of manually gated immune cell subsets in the validation cohort with significance indicated (blue, *P* < 0.05). (**F**) MDS plot of samples based on manually gated features showing significant group separation (PERMANOVA *P* < 0.0001, *R*^2^ = 0.35).

To identify minimal immune signatures distinguishing rural and urban populations across datasets, we performed a multi-omics discriminant analysis using DIABLO (*46*). This model—using features associated with B cells, NK cells, CD4^+^ T cells, and monocytes—resulted in distinct sample clustering (Fig. 7A) with a mean overall balanced accuracy exceeding 90% (Fig. 7B). Urban Senegalese individuals had the lowest class-wise accuracy, possibly due to their intermediate position between rural Senegal and urban Netherlands dwellers in the consensus embedding. Cross-validation–based permutation analysis supported the model’s robustness (*P* < 0.001; Fig. 7B).

Having shown the accuracy of the above DIABLO model, we next explored the features selected by this model (Fig. 7C). We found that CD161^+^CD4^+^T cell clusters were among the top 10 discriminating features for rural individuals and G6PD levels in CD4^+^T and NK cells. IL-1β^+^CD38^+^ monocytes, CD38^+^ naïve CD4^+^T cells, and Cytc levels in NK cells were among the key features in distinguishing urban individuals. The model also identified additional clusters, such as CD45RA^+^ T_reg_ and PD-1^+^ T_H_1 cells, that were enriched in urban and rural settings, respectively. These findings confirmed our single dataset analysis results and revealed additional distinguishing features.

To test the generalizability of these immune signatures, we analyzed mass cytometry EXV and PMA datasets from rural and urban Indonesians (*4*). We gated 16 immune cell subsets from these datasets (Fig. 7D) and found that 11 of the 16 signatures from the Senegalese cohort were statistically significant in this independent population, showing consistent trends (Fig. 7E). The validated subsets included those expressing checkpoint markers (CTLA-4^+^PD-1^+^CM CD4^+^T, PD-1^+^ T_H_1, and PD-1^+^CD38^+^CD56^dim^CD16^+^ NK), those indicating heightened activation (CD56^+^ monocytes, CD161^+^T_H_2), and those with enhanced pro-inflammatory profile (TNF^+^CD27^+^CD161^+^CD4^+^T). Using the frequencies of the 16 manually gated subsets as input, multidimensional scaling showed significant separation between residence groups (*R*^2^ = 0.35, PERMANOVA *P* < 0.0001; Fig. 7F).

These results demonstrate that the key rural-to-urban immune signatures initially identified in Senegalese populations can also be found in an independent cohort from a distinct geographical region and ancestry, indicating a shared immunological adaptation to rural-urban environments across diverse populations.

### Proinflammatory cellular cytokine responses were differentially affected by metabolic enzyme inhibition

**Fig. 8.**
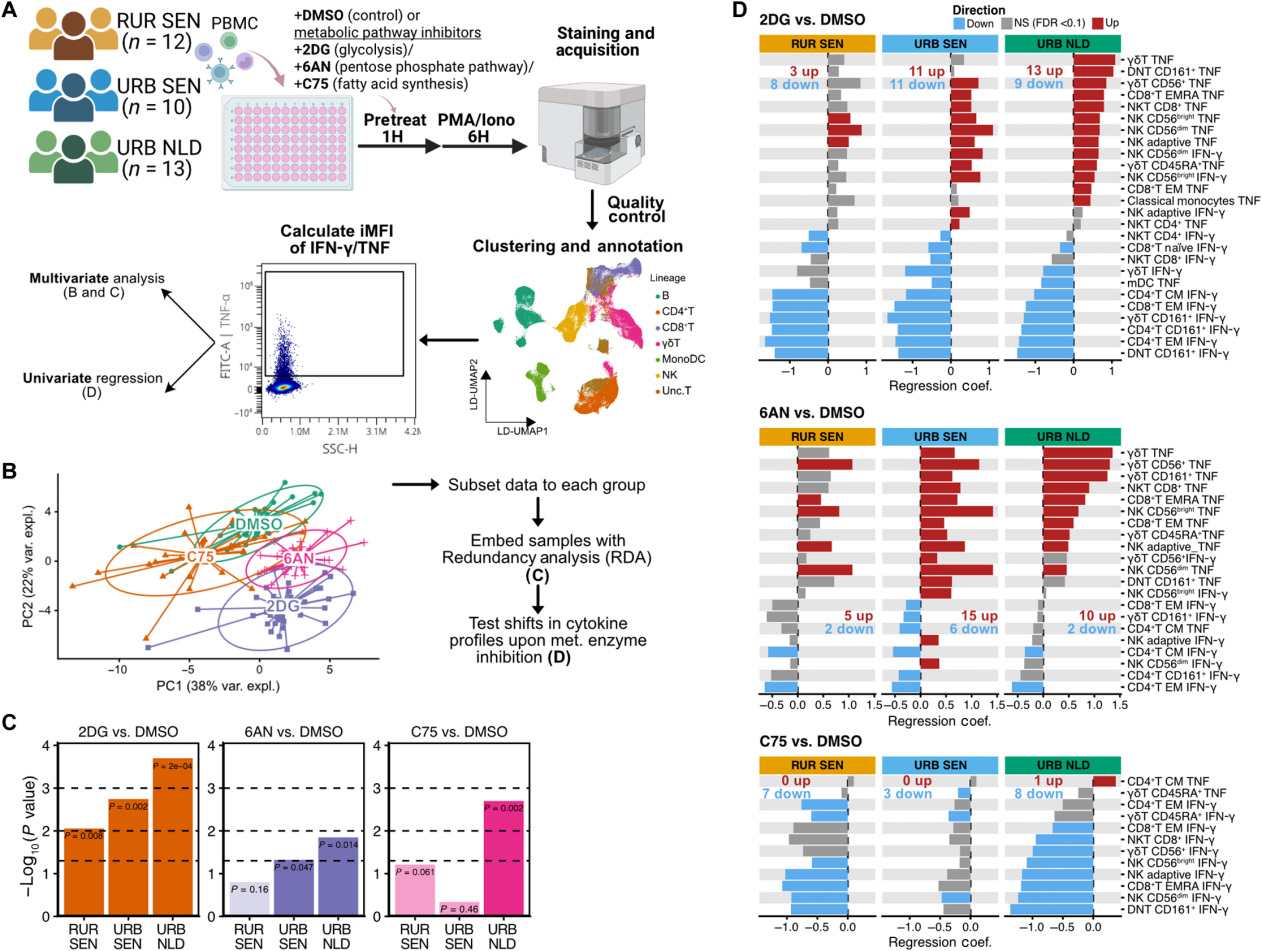
Differential impact of metabolic enzyme inhibition on cellular cytokine responses across populations. (**A**) Experimental workflow: PBMCs (*n* = 12 RUR SEN, *n* = 10 URB SEN, and *n* = 13 URB NLD) were treated with metabolic enzyme inhibitors or DMSO control before PMA/ionomycin stimulation. The iMFI of cytokines (IFN-γ and TNF) was calculated for each immune cell subset. iMFI was calculated by multiplying frequency of cytokine-positive cells relative to its parent subset with mean fluorescence intensity of cytokine-positive cells. This figure was created using BioRender. (**B**) Principal components analysis (PCA) plot of cytokine responses following metabolic inhibition. iMFI obtained from (A) from all subset subsets and cytokine pairs was used as input for PCA. Within-individual *z* scores were calculated to take into account repeated measures. Points represent individuals, colored by pretreatment conditions. (**C**) Significance (−log_10_ *P* values) of cytokine response shifts after metabolic inhibition versus DMSO control for each residence groups (RDA permutation tests, *n* = 5000). (**D**) Effect sizes of iMFI changes for 2DG, 6AN, or C75 pretreatments compared to DMSO control. Bars show regression coefficients from linear-mixed models. Colors indicate significance and direction: gray (FDR ≥ 0.1), red (FDR < 0.1, increase), and blue (FDR < 0.1, decrease). Only subset-cytokine pairs with FDR < 0.1 in at least one group per inhibitor are shown. 2DG, 2-deoxy-d-glucose; 6AN, 6-aminonicotinamide; mDCs, myeloid dendritic cells; NK, natural killer cells; γδT, gamma delta T cells; NKT, natural killer T cells; DNT, double negative (CD4^−^CD8^−^) T cells; CM, central memory; EM, effector memory; EMRA, terminally differentiated effector memory cells re-expressing CD45RA; FITC, fluorescein isothiocyanate.

Having established the variation in immune profiles across the rural-urban gradient and its potential link to metabolic rewiring, we investigated the differential impact of metabolic pathway perturbations on proinflammatory cytokine responses upon activation. We stimulated cells from an independent rural and urban Senegalese cohort from the same areas with PMA in the presence or absence of inhibitors of metabolic enzymes that are part of metabolic pathways known to play a key role in supporting inflammatory immune cell responses: 2-deoxy-d-glucose (2DG) (glycolysis), 6-aminonicotinamide (6AN) (PPP), or C75 (FAS) (Fig. 8A). Using spectral flow cytometry, we identified 23 immune subsets (fig. S8, A and B) and quantified cytokine production using integrated mean fluorescence intensity (iMFI) for downstream analyses.

Principal component analysis showed that inhibition of metabolic pathways altered proinflammatory cellular cytokine responses (Fig. 8B and fig. S8C). We next performed redundancy analysis (RDA) to quantify overall shifts in cytokine responses following metabolic perturbations across populations. Shifts in cytokine responses after 2DG and 6AN pretreatment followed a rural-urban gradient with the least responses seen in rural Senegalese and the most in Dutch individuals (Fig. 8C)—Significant response to metabolic perturbation in rural Senegalese was seen only following 2DG (*P* = 0.008).

We analyzed how metabolic pathway inhibitors affected cytokine-producing cell subsets across populations (Fig. 8D and fig. S8D). The glycolysis inhibitor 2DG generally enhanced TNF production while reducing IFN-γ production, with effects most pronounced in Dutch individuals, followed by urban and rural Senegalese (Fig. 8D, top). The PPP inhibitor 6AN increased TNF production similarly to 2DG but affected IFN-γ in fewer subsets, with the strongest shifts seen in urban Senegalese and the weakest in rural Senegalese individuals (Fig. 8D, middle). The FAS inhibitor C75 mainly suppressed IFN-γ response, most notably in Dutch participants, followed by rural and urban Senegalese (Fig. 8D, bottom). Together, these findings underscore the differential impact of metabolic perturbations on proinflammatory responses across populations, suggesting distinct metabolic adaptations along the rural-urban gradient.

## DISCUSSION

Our extensive multi-omics analysis reveals distinct immunological signatures across the rural-urban gradient in understudied populations of the Global South. We demonstrate that the rural Senegalese individuals exhibited altered IgG glycosylation profiles, adaptive NK cells with reduced responsiveness to accessory cytokines, and proinflammatory CD11c^+^ B cells, CD56^+^ monocytes, and CD161^+^CD4^+^T cells. By validating key signatures in an independent Indonesian cohort, we provide evidence for shared immunological adaptations to environmental exposures across diverse geographical settings. Notably, we uncovered differential sensitivities to metabolic perturbations among populations, with rural Senegalese exhibiting the least response. Collectively, these findings provide unprecedented details into the cellular and humoral immune compartments modulated by rural-urban gradient, highlighting critical considerations for developing health interventions for populations in the Global South.

Reduced IgG Fc galactosylation in rural individuals is likely driven by higher pathogen exposure including parasitic helminths common in low- and middle-income countries (*18*, *47*). Reduced IgG galactosylation has been linked to its proinflammatory profile in aging (*48*) and inflammatory disorders (*17*, *18*, *49*). This glycosylation profile has been associated with suppressing the inhibitory IgG receptor FcγRIIB (*50*) and lowering the C1q complement activation threshold (*51*). Therefore, it is likely that such a glycosylation pattern may prove beneficial for protecting against the higher infection pressure in rural settings.

Our findings suggest an interplay between altered IgG glycosylation patterns and proinflammatory CD11c^+^ B cells (*52*). The transcriptomic profiling of these cells suggests regulation by RelA, a subunit of NF-κB, which has been implicated in inflammaging and TNF production (*53*). Notably, we observed elevated expression of glycosyltransferase-encoding genes in CD11c^+^ B cells, which have been proposed as potential precursors to antibody-secreting cells (*26*, *27*). Previous studies have demonstrated that modulation of glycosylation enzymes in IgG-secreting B cell lines can affect IgG glycosylation (*54*), but whether CD11c^+^ B cells contribute to this process in vivo is unknown. To elucidate any potential relationship between CD11c^+^ B cells and IgG glycosylation, further mechanistic studies are warranted. However, the rarity of CD11c^+^ B cells presents a technical challenge, requiring the development of robust B cell expansion techniques (*55*, *56*).

The NK cell compartment in rural settings showed a more differentiated phenotype, characterized by the enrichment of NKG2C^+^ adaptive NK cells, often associated with CMV seropositivity (*32*, *34*, *57*). Although these cells showed reduced IFN-γ response to IL-12/IL-18 stimulation (*33*, *34*), this likely reflects functional specialization, with NKG2C^+^ NK cells shifting from cytokine-dependent to receptor-mediated activation against HLA-E–expressing targets (*35*, *58*). This shift may explain rural individuals’ reduced response to MPL-A, which relies on cytokine-mediated NK cell activation (*59*, *60*). We hypothesize that this finding may partly explain the suboptimal efficacy of vaccines in African populations, such as the RTS/AS01 malaria vaccine, of which MPL is a crucial component (*61*).

Another factor that drives immune variation across the urban/rural gradient is the enrichment of CD161^+^CD4^+^T cells with enhanced proinflammatory cytokine production capacity in rural-living individuals. This subset has been previously linked to protective immunity against malaria (*62*) and improved cancer outcomes (*63*). The enrichment of these cells in rural settings may represent a functional adaptation to high infectious pressures (*4*, *62*), consistent with the findings that inflammatory conditions can increase CD161^+^CD4^+^T cell abundance (*64*). Notably, CD161^+^CD4^+^T cells from rural individuals exhibited enhanced proinflammatory profiles, as characterized by enrichment of cytokine-producing cells following activation, increased expression of IFN-related genes (*IFIT3* and *IFI44L*), higher CD151 activation marker, and decreased BTLA (CD272) inhibitory receptor expression (*38*).

Monocytes from rural individuals exhibited a heightened proinflammatory profile, characterized by increased expression of HLA-DR, CD56, CD64, and CD86, along with elevated TNF production. The heightened activation is accompanied by *HLA-ABC* up-regulation to support antigen presentation capacity (*44*). This proinflammatory phenotype aligns with observations of CD56^+^ and CD64^+^ monocytes in inflammatory disorders (*39*, *43*, *65*). Higher *SERPINB10* expression in monocytes of rural individuals suggests an adaptive mechanism to withstand TNF-induced apotosis^47^, which could occur due to the inflammatory environment in pathogen-rich rural settings (*45*, *66*). The self-limiting modulatory effect of TNF on IL-12 production (*67*) provides an alternative explanation for the reduced IFN-γ production by NK cells in response to MPL-A stimulation in rural settings, highlighting a potential mechanism linking monocyte activation to altered NK cell function.

We observed a shift toward a proinflammatory immune profile in the blood of rural Senegalese compared to urban Senegalese and urban Dutch individuals. Given the growing recognition of metabolism in regulating immune function at both cellular and organismal levels, we investigated metabolic protein expression across groups. We found a consistent increase in the expression of PPP enzyme G6PD and FAS enzyme ACC1, accompanied by a decrease in the oxidative phosphorylation enzyme Cytc across multiple immune cell lineages in rural settings. These changes suggest systemic causes underlying this metabolic adaptation, potentially linked to diet and pathogen exposure (*68*, *69*). Increased FAS, supported by reduced form of nicotinamide adenine dinucleotide phosphate derived from the PPP (*70*, *71*), is associated with immune senescence, characterized by cell-cycle arrest and proinflammatory properties (*72*). The reduction in Cytc aligns with dysfunctional mitochondria observed in senescent cells (*72*). While senescence was originally associated with aging, other factors, such as chronic pathogen exposure, can accelerate immune aging (*8*, *73*), potentially explaining the altered metabolic phenotype in rural individuals. Increased FAS is also a metabolic feature of trained immunity (*74*), supporting the link between metabolic changes and immune adaptation in these populations.

We investigated the functional impact of metabolic differences on cytokine production across the rural-urban gradient by inhibiting metabolic pathways during cell stimulation. Remarkably, the Dutch individuals showed the most sensitivity to inhibition, followed by urban Senegalese, with rural Senegalese showing the fewest changes. FAS inhibition particularly affected the Dutch individuals, decreasing cytokine expression. While the link between FAS and proinflammatory immune function is well established (*75*), the mechanism by which it influences acute cytokine production during short-term stimulation remains unclear. We observed increased cytokine production in NK cells following glycolytic inhibition. This finding contrasts with previous reports on the reliance of NK cells on glycolysis for activation and IFN-γ production (*76*–*79*). Possibly, NK cells may be indirectly affected by metabolic inhibition due to interactions with other cell types present in our peripheral blood mononuclear cell (PBMC) cultures. Further investigation into these specific interactions is warranted. Urban Senegalese populations were most affected by PPP inhibition and least affected by FAS inhibition. Our data revealed altered metabolic sensitivities in immune cells along a rural-urban gradient. The differential sensitivity to metabolic perturbations was observed across populations, with rural Senegalese showing the least responsiveness, suggesting distinct dependencies on the examined metabolic pathways to support proinflammatory responses.

Our observation of elevated inflammatory signatures in rural settings seemingly contradicts evidence linking western lifestyles to inflammatory diseases (*21*, *80*, *81*). This discrepancy might reflect distinct inflammation triggers, with rural signatures likely representing adaptations to pathogen exposure (*8*, *20*), unlike the chronic low-grade metabolic inflammation observed in urban settings (*21*). Supporting this, rural Senegalese showed elevated cytokines linked to pathogen-driven inflammation (C5a and IL-8) (*82*), whereas urban Dutch showed elevated CCL2/MCP-1, characteristic of metabolic inflammation (*83*).

Our study provides a detailed characterization of immune and metabolic variations in rural-to-urban settings in an understudied population. However, this study had several limitations, including small sample size, generalizability, categorization of urbanization, lack of rural Dutch individuals, bias toward early-phase cytokines, and cell-cell interaction yet to be identified. We made a trade-off of analyzing more modalities over more individuals. Despite the relatively small numbers of individiuals, the proinflammatory immune signatures found in rural dwellers were found across several biological layers and readouts. Moreover, we validated key signatures in an ancestrally and geographically distinct cohort in Indonesia, indicating the robustness of our findings. Our findings primarily represent rural settings with low socioeconomic status where helminth infections remain endemic—a reality for many, but not all, rural LMIC populations (*84*). Considering helminths, we showed that the immune profile of individuals with no current infection, evidenced by highly sensitive assay, were comparable to those who were tested positive. With respect to urban Senegal settings, their comparable body mass index (BMI) values to those living in rural settings likely reflect our exclusion of chronic conditions, including diabetes, often associated with elevated BMI. However, it should be noted that Sahelian populations, including Senegal, generally have lower BMI compared to other African regions (*85*). Although we categorized areas as rural or urban for simplicity, continuous measures capturing additional lifestyle and environmental nuances might provide a more precise assessment of urbanization (*86*). Moreover, inclusion of rural Dutch individuals might help separating genetic and environmental effects in shaping the immune system; however, evidence suggests subtle difference between rural and urban populations in high-income countries (*18*), highlighting the need for further investigation. Taking into account that the temporal kinetics of cytokine production vary between cytokines and across cell types, our results could be biased toward early-phase cytokines and therefore must be interpreted accordingly (*87*). Last, although we could identify immune cell subsets driving the variation along the rural-to-urban gradient, the functional interaction among the subsets remains largely unclear.

In conclusion, our study provides a comprehensive resource of immune and metabolic variations across the rural-urban gradient in understudied populations. We have identified key cellular subsets and metabolic features driving this variation. Expanding this study to have a broader representation across locations, ancestries, and age groups will be crucial for distinguishing between inflammation associated with aging versus rural living. Our findings provide immunologic and metabolic targets for mechanistic studies to better understand immune regulatory networks in rural populations. This has particular applications for improving vaccine responsiveness and understanding differential disease outcomes across regions and rural-to-urban gradients. Ultimately, this work underscores the need to consider the environmental context in immunological research and public health strategies, particularly in regions undergoing rapid urbanization.

## MATERIALS AND METHODS

### Description of study areas

The study was conducted across diverse geographical and socioeconomic settings: rural and urban areas in Senegal (Western Africa) and an urban area in the Netherlands (Western Europe). Rural Senegalese participants were recruited from Pakh and Richard Toll, villages in northern Senegal characterized by large family units and subsistence farming. Despite having access to clean water, frequent contact with river water occurs due to agricultural activities. Urban Senegalese participants were selected from Dakar, the capital city, representing individuals of higher socioeconomic status who predominantly adopt western lifestyles with improved sanitation and consistent access to clean water.

Rural areas in the Senegal River basin, such as Richard Toll, have historically exhibited high prevalence rates of schistosomiasis, with some regions reporting rates of up to 80% or higher (*88*). In contrast, Dakar is considered non-endemic for this parasitic infection (*88*, *89*). Recent retrospective studies conducted in Dakar have revealed overall parasite prevalence rates ranging from 18.9 to 26.4% for various intestinal parasites, including *Blastocystis* sp., *Entamoeba coli*, *Endolimax nana*, *Giardia intestinalis*, and *Entamoeba histolytica* (*90*, *91*). However, these prevalence rates in Dakar may be overestimated due to potential selection bias, as the data were derived from patients undergoing diagnostic testing for parasitic infections.

Senegal’s population includes Wolof (43.3%), Pular (23.8%), and Serer (14.7%) ethnic groups with substantial cultural overlap and frequent intermarriage. The Wolof, the largest ethnic group in Senegal, predominantly inhabits the western and northern regions of the country. Notably, ethnic distinctions are increasingly blurred through cultural admixture, particularly in rural areas.

### Study design

This study was approved by the ethics committee of Cheikh Anta Diop University of Dakar, Senegal (reference no. 0339/2018/CER/UCAD) and Leiden University Medical Center, the Netherlands (CCMO reference no. NL66287.058.18 and OMON trial register: NL-OMON48968). Written informed consent was obtained from participants before the study.

Participants were recruited through local health care centers in rural and urban sites in Senegal and through Leiden University Medical Center in the Netherlands from November 2018 to August 2019. All participants underwent thorough medical examinations by clinicians who verified the absence of any clinical signs of current infection or chronic inflammatory disease. Each participant completed comprehensive questionnaires documenting demographic information, socioeconomic status, dietary patterns, lifestyle factors, health-seeking behaviors, and medical history. Inclusion criteria are as follows: residence in the area for ≥10 years, age 18 to 40, no chronic illnesses including diabetes and hypertension, and negative malaria (after thick smear and malaria rapid test), *Schistosoma* species (after Kato-Katz test on the feces and urine filtration test using 12-μm-pore size filters) as well as *Ascaris lumbricoides*, *Trichuris trichiura*, and hookworm following microscopic examination of the feces. Serum CAA analysis was performed using the upconverting particle-lateral flow CAA (UCP-LF SCAA500) assay as described previously (*92*).

Participants meeting the inclusion criteria were recruited from three locations: rural Senegal (*n* = 72, from Pakh and Richard Toll), urban Senegal (*n* = 46, from Dakar), and urban Netherlands (*n* = 28). Most Senegalese individuals were Wolof (94.7% in urban cohort based on surname analysis; predominantly Wolof in rural Pakh village). Rural versus urban classifications were based on objective criteria from questionnaire data (household size, animal contact, housing conditions, and amenities; table S2), extending beyond geographical designations. All urban Senegalese participants resided in the same area in Dakar considered to be of high socioeconomic status. For this study, a subgroup of individuals was randomly selected, matching for age and sex.

For this exploratory multi-omics study, sample size was based on prior work showing sufficient power to detect meaningful immune differences across rural-urban populations with around 10 individuals per group (*4*, *16*). To assess generalizability, findings from the Senegalese cohort were compared to published mass cytometry data from matched rural-urban groups in Indonesia (*4*).

### Sample collection and PBMC cryopreservation

To ensure comparable sample quality across the study sites, we implemented standardized procedures in all sites. Technical staff received centralized training at Leiden University Medical Center and followed standardized operating procedures for sample collection and processing. Venous blood was collected in heparin, EDTA, and dry tubes for cell isolation, full blood count, and serology tests.

PBMCs were isolated from heparinized blood within 4 hours using Ficoll density gradient centrifugation. Cells were cryopreserved in freezing medium and stored in liquid nitrogen. Freezing medium consisted of penicillin (100 U/ml; Gibco), streptomycin (100 U/ml; Sigma-Aldrich), 1 mM pyruvate (Sigma-Aldrich), 2 mM glutamate (Sigma-Aldrich), and 10% dimethylsulfoxide (Merck) in heat-inactivated fetal calf serum (Invitrogen). The cryopreserved PBMCs collected in the field were shipped in a liquid nitrogen dry vapor shipper to Leiden, the Netherlands, for centralized analysis. Temperature monitoring systems were used during sample transport from Senegal to the Netherlands to document and ensure maintenance of optimal storage conditions. To minimize technical variation, all samples were processed and analyzed in Leiden University Medical Center. All PBMC samples included in this study showed greater than 85% viability.

### CMV serology

Serum anti-CMV IgG titers were determined using a semi-quantitative VIDAS CMV IgG assay kit (Vitek ImmunoDiagnostic Assay System, bioMérieux) following the manufacturer’s protocol. Samples with ≥6 arbitrary units/ml were considered CMV seropositive.

### IgG Fc glycosylation analysis by mass spectrometry

IgG Fc-glycopeptides were profiled for 28 individuals (GLY dataset). IgG antibodies were affinity-purified using Protein G Sepharose beads. Purified IgG was trypsinized, and the resulting glycopeptides were analyzed using liquid chromatography–mass spectrometry (LC-MS) (*93*). Lacy Tools were used to quantify glycopeptides, and IgG subclass-specific Fc glycosylation profiles were determined, resulting in the quantification of 24 and 14 glycopeptides for IgG1 and IgG2, respectively (table S10). Note that the IgG3 glycopeptide sequence shows allotype variation in the amino acid at the position N-terminal of the Asn^227^, causing a mass that is identical to the IgG4 sequence (EEQYNSTFR, predominant allotype in Asian and African populations). Because of this allotype variation, IgG3/IgG4 glycopeptides cannot be discriminated in this study and thus excluded from downstream analysis (*94*, *95*). Glycosylation traits such as fucosylation, galactosylation, bisection, sialylation, and the amount of sialic acid linked to a galactose residue (SA/gal) were calculated for the Fc glycosylation sites of each subclass. IgG fucosylation levels were determined by dividing the sum of all fucosylated N glycopeptide species (G0F, G1F, G2F, G1FN, G1FS, and G2FS) by the total of all glycan species. Galactosylation levels were calculated by using the formula [0.5 × (G1F + G1FN + G1FS + G1) + G2F + G2FS + G2 + G2S/Σ (all glycan species)]. Sialylation was determined as 0.5 × (G1FS + G2FS) + G2F1S2/Σ (all glycan species). Bisection was calculated by dividing G1FN by the sum of all glycan species.

### Multiplex immunoassay

The R&D Systems Luminex Discovery Assay Human Premixed Multi-Analyte Kit was used to measure 19 cytokines in plasma: CCL3, CCL8, CCL24, complement component C5a, CXCL10, HGF, IL-3, IL-8, IL-18, vascular endothelial growth factor, CCL2, CCL7, CCL20, CXCL9, CXCL13, IFN-γ, IL-6, IL-10, and S100A8. Manufacturer’s instructions were followed, except half of the suggested reagent volumes were used. Briefly, plasma samples were spun at 16,000*g* for 5 min at 4°C. Then, 25 μl was mixed with 25 μl of calibrator diluent RD6-52 and incubated with 25 μl of microparticle cocktail for 2 hours at room temperature (RT) on a plate shaker at 800 RPM.After three washes, 25 μl of biotin-antibody cocktail was added and incubated for 1 hour at room temperature (RT) on a plate shaker. Then, another three washes were performed and 25 μl of streptavidin-phycoerythrin was added and incubated for 30 min at RT on a plate shaker. After three final washes, the microparticles were resuspended in 80 μl of wash buffer, incubated for 2 min at RT while shaking and immediately read on a Bio-Rad Bio-Plex 200 system at 50 counts per region and default settings. Analytes that were not detected (lower than the limit of detection) in more than 40% of samples were excluded for analysis. Measurement values below the lower limit of detection (LLOD) were imputed with half the LLOD values.

### Mass cytometry analysis

Ex vivo immunophenotyping (EXV dataset, *n* = 37) and analysis of cellular cytokine responses following stimulation with PMA/ionomycin (PMA dataset, *n* = 37) or MPL-A (MPL dataset, *n* = 37) were performed using mass cytometry.

#### Cell culture

To investigate differential immune responses within rural and urban populations of Africa and Europe, PBMCs were cultured with either MPL vaccine adjuvant or PMA and ionomycin. Briefly, 3 × 10^6^ PBMCs were cultured for 6 hours with either 10% fetal calf serum (FCS)/RPMI as control, MPL (2.5 mg/ml; Invivogen), or PMA (100 ng/ml; Sigma-Aldrich) and ionomycin (1 μg/ml; Sigma-Aldrich). Brefeldin A (10 μg/ml; Sigma-Aldrich) was added for the final 4 hours.

#### Staining procedure

Samples were randomized to avoid batch effects. Antibody panels are detailed in tables S11 and S12. Antibodies were either purchased or conjugated using 100 μg of purified antibody combined with the MaxPar X8 Antibody Labelling Kit (Fluidigm, South San Francisco, CA, USA), following the manufacturer’s protocol. Conjugated antibodies were stored in 200 μl of antibody stabilizer phosphate-buffered saline (PBS) (Candor Bioscience, GmbH, Wangen, Germany) at 4°C.

Cryopreserved PBMCs were thawed in 50% FCS/RPMI medium at 37°C and washed twice with 10% FCS/RPMI. Three million cells were used for EXV (mass cytometry panel 1; table S11). Staining was performed using MaxPar Nuclear Antigen Staining Protocol V2 (Fluidigm). Cells were washed with MaxPar staining buffer and centrifuged for 5 min at 300*g* in 5-ml eppendorf tubes. Then, cells were incubated with 1 ml of 500× diluted 500 μM Cell-ID Intercalator-103Rh (Fluidigm) in staining buffer at room temperature for 15 min to identify dead cells. After washing with staining buffer, cells were incubated with 5 μl of Human TruStain FcX Fc-receptor blocking solution (BioLegend, San Diego, CA, USA) and 40 μl of staining buffer at room temperature for 10 min. Next, 5 μl of freshly prepared surface antibody cocktail was added, and the mixture was incubated at room temperature for another 45 min. Subsequently, cells were washed twice with staining buffer, fixed, and permeabilized using eBioscience FOXP3/Transcription factor staining buffer set (eBioscience, catalog no. 00-5523-00). After cells were incubated with 1 ml of freshly prepared Fix/Perm working solution (prepared according to the manufacturer’s instructions) for 45 min, cells were washed twice with 1× permeabilization buffer at 800*g* for 5 min. Next, 50 μl of intranuclear antibody cocktail was added to 50 μl of cells resuspended in 1× permeabilization buffer and incubated for 30 min at room temperature. Following incubation, cells were washed once with 1× permeabilization buffer and twice with staining buffer before being stained with 1 ml of 1000× diluted 125 μM Cell-ID Intercalator-Ir (Fluidigm) in MaxPar Fix and Perm buffer (Fluidigm) at 4°C overnight to stain all cells. After three washes with staining buffer and centrifugation at 800*g*, stained cells were stored as pellets at 4°C and analyzed within 2 days.

Cells were stained using mass cytometry panel 2 to assess cellular cytokine response following 6 hours of culture in medium, MPL, or PMA/ionomycin (table S12). Staining was based on MaxPar Cytoplasmic/Secreted Antigen Staining Protocol V3. While surface staining was performed as described above, cells were fixed by incubating them with 1 ml of freshly prepared 1× MaxPar Fix I buffer (Fluidigm) for 20 min at room temperature. Next, cells were washed 3× with MaxPar Perm-S buffer (Fluidigm), and 50 μl of cytokine antibody cocktail was added to 50 μl of cell suspension and incubated for 40 min at room temperature. Then, cells were washed 3× with staining buffer and stained with Cell-ID Intercalator-Ir, as described above. Stained cells were stored as pellets at 4°C and analyzed within 2 days.

#### Data acquisition

Samples were randomly measured per individual to avoid bias; however, samples from the same individual were stained and measured together. Samples were analyzed using a HeliosTM mass cytometer (Standard BioTools), which was automatically tuned according to the manufacturer’s recommendations.

Before measurement, cells were counted, washed with Milli-Q water, passed over a cell strainer, and brought to a concentration of 1.0x10^6^ cells/mL with 10% EQ Four Element Calibration Beads (Standard BioTools) in Milli-Q water. Mass cytometry data were acquired and analyzed on the fly using the dual-count mode and noise reduction. Channels for intercalators (103Rh, 191Ir, and 193Ir), calibration beads (140Ce, 151Eu, 153Eu, 165Ho, and 175Lu), and background/contamination (133Cs, 138Ba, and 206Pb) were acquired. After data acquisition, a mass bead signal was used to normalize short-term signal fluctuations with the reference EQ passport P13H2302 during each experiment. When applicable, normalized FCS files were concatenated using Helios software without removing beads.

#### Debarcoding

Debarcoding of pooled samples was performed using the debarcoder utility in Helios software with default parameters.

#### Quality control

FlowJo V10 for Mac (FlowJo LLC, Ashland, OR, USA) was used to gate out beads, DNA staining, and live CD45^+^ cells. Additional quality control using Gaussian parameters was performed using the CyTOF QC R package. Signal stability over acquisition time was performed using the PeacoQC R package with parameters recommended for mass cytometry data (*96*).

#### Batch effect evaluation and correction

Batch effect was qualitatively evaluated using fast Fourier transform-accelerated interpolation-based t-distributed stochastic neighbor embedding (FI-tSNE) with default parameters. Batch effect was found in ex vivo–stimulated cells (panel 2) and corrected using CytoNorm (*97*). FI-tSNE embedding showed confirmed a markedly reduced batch effect after correction.

#### Unsupervised clustering

Clustering was performed in two steps. First, FlowSOM (*98*) was performed on all available markers using Euclidean distance, 10 × 10 grid, and rlen 100. Resulting clusters were aggregated into known immune cell lineages based on median marker expression. Second, FastPG (*99*) (*k* = 50, Euclidean distance) was used to subcluster each immune-cell lineage using only biologically relevant markers as input. Clusters were then merged and annotated guided by expert knowledge.

#### Supervised dimensionality reduction

For visualization of mass cytometry data, the Uniform Manifold Approximation and Projection (UMAP) algorithm was performed using linear discriminant analysis (LDA) initialization (*100*). Cells (5000 to 10,000) were subsampled per cluster for LDA, with cluster labels as the response variable. The first two resulting components (LD1 and LD2) were used to initialize UMAP embedding, facilitating immune cell cluster separation.

#### Statistical analysis

DA analysis was performed using arcsine square root–transformed cluster frequencies. Residence groups were coded as ordered factors (RUR SEN > URB SEN > URB NLD or RUR IDN > URB IDN > URB NLD). Lineage- and subset-level analyses used generalized least squares (nlme R package), including age and sex as covariates and allowing unequal variances per group. Linear trend test (orthogonal polynomial contrast) and pairwise contrast *P* values (Tukey’s correction) were obtained using the emmeans R package. Cluster-level analysis was performed using the limma package in R (*101*), with trend test *P* values obtained using the topTable function and corrected for multiple testing using the Benjamini-Hochberg method. Considering our aim to determine features showing ordered linear increase from RUR SEN to URB SEN to URB NLD and vice versa, features with significant linear component of orthogonal polynomial contrasts (FDR < 0.1) and nonsignificant quadratic trend component were considered as significant (*102*).

### Spectral flow cytometry

Spectral flow cytometry was performed to obtain MET levels (MET dataset, *n* = 27) and to determine changes in cytokine responses with or without metabolic enzyme inhibition in an independent cohort.

### Metabolic enzyme analysis

#### Staining procedure

Antibodies and dilutions are listed in table S13. Antibodies against metabolic targets were conjugated in-house using Lightning Link kits (Abcam). One million thawed cells were incubated with viability dye and Fc block in PBS for 15 min on ice and then stained for surface markers in fluorescence-activated cell sorting buffer for 30 min on ice. Cells were fixed with Foxp3 fixation/permeabilization buffer (eBioscience) and stained for remaining surface and intracellular targets. All staining was performed in buffer containing 1× Brilliant Stain Buffer Plus (566385, BD Biosciences) and TrueStain Monocyte Block (426103, BioLegend). Intracellular targets were stained in 1× permeabilization buffer with Fc block for 2 hours at 4°C. Single-stained reference cell controls were simultaneously stained in parallel. Cells were acquired using a five-laser Cytek Aurora spectral flow cytometer.

#### Data analysis

Samples were unmixed using SpectroFlo version 3 and analyzed on the OMIQ.ai web platform. Fluorescence parameters were scaled using conversion factors ranging from 5000 to 20,000. Cell subsets were manually gated, and the 80th percentile of metabolic target expression levels was calculated for downstream analysis.

### Metabolic enzyme inhibitor analysis

#### Staining procedure

Antibodies and dilutions are listed in table S14. Thawed PBMCs (5 × 10^5^ cells per well) were incubated for 1 hour with 40 μM C75 (Tocris, catalog no. 2489), 100 μM 6AN (Sanbio, catalog no. 10009315-250), 10 mM 2DG (Sigma-Aldrich, catalog no. D8375), or dimethyl sulfoxide (DMSO) control. Cells were then stimulated with PMA/ionomycin for 2 hours, followed by Brefeldin A for 4 hours. Cells were stained with live/dead marker, monocyte blocker, and Fc block at room temperature for 15 min. Cells were then stained with 30 μl of surface antibody mix for 30 min on ice, fixed with 1.85% formaldehyde for 10 min, permeabilized, and lastly stained with 30 μl of intracellular target mix for 30 min at room temperature. Samples were acquired using a Cytek Aurora spectral flow cytometer.

#### Data analysis

Samples were unmixed using SpectroFlo version 3 and analyzed on the OMIQ.ai web platform. Fluorescence parameters were asinh-transformed with a cofactor of 6000. After quality control gating (doublet and dead cells removal), data were batch-corrected using cyCombine in R (*103*), clustered using SOM with consensus clustering, and merged to subset-level annotations. The iMFI (defined as frequencies of cytokine^+^ cells relative to parent subset × mean fluorescence intensity of cytokine^+^ cells) was calculated for IFN-γ and TNF production, except in B cells and monocytes for IFN-γ.

#### Statistical analysis

Principal components analysis was performed using the mixOmics R package with within-individual scaling. The significance of group centroid distances was performed on principal component coordinates using the adonis2 function (vegan R package) with method = “euclidean.” Redundancy analysis (RDA) used the rda function (vegan R package). Regression analysis used a linear mixed model (lme function, nlme R package) with restricted maximum likelihood and batch ID and individual ID as random effects.

### CITE-seq

#### Staining procedure

One million thawed PBMCs were washed in cell staining buffer (BioLegend, catalog no. 420201) and stained with Fc block for 15 min on ice. For sample multiplexing, 1 μl of TotalSeq-A hashtag (BioLegend) was added to each sample and incubated on ice for 30 min. After washing, 200,000 cells from each sample were pooled and stained with TotalSeq-A universal cocktail (BioLegend, catalog no. 399907) following the manufacturer’s protocol. The pooled sample was washed and filtered using a Flowmi cell strainer (Bel-Art, catalog no. H13680-0040).

#### Single-cell library preparation and sequencing

Single-cell RNA sequencing was performed using the 10X Genomics Chromium Next GEM Single Cell 3′ Kit v3.1 (PN-1000268) and 10X Genomics Chromium Next GEM Chip G Single Cell Kit (PN-1000120) according to the manufacturer’s instructions (User Guide CG000315 Revision A) with the following modifications. During the cDNA amplification step, two additional primers were added to increase the yield of the CITE-seq and hashtag barcodes [antibody derived-tags (ADT) polymerase chain reaction (PCR) additive primer (CCTTGGCACCCGAGAATTCC) and HTO PCR additive primer (GTGACTGGAGTTCAGACGTGTGCTC)]. After cDNA amplification, a 0.6X SPRI bead selection was performed to separate the CITE-seq and hashtags barcodes [<180 base pair (bp)] from the cDNA fraction (>300 bp). The CITE-seq and hashtag libraries were constructed from the <180-bp fraction (*104*). The libraries were pooled and sequenced on NovaSeq 6000 (Illumina) using a 200-cycle kit S4 flow cell with v1 chemistry. Fastq files were generated using cellranger mkfastq, and count tables were generated using Cell Ranger multi version 5.0.0 (10X Genomics) with human genome build GRCh38 as the reference genome (10X Genomics annotation file refdata-gex-GRCh38–2020-A).

#### Quality control

Cell-level quality control used three metrics: (i) gene counts per cell, (ii) detected genes per cell, and (iii) percentage of mitochondrial genes per cell. Low-quality cells were excluded using an adaptive threshold of three median absolute deviations (*105*). Genes expressed in at least three cells were retained. Cells were demultiplexed using the HashedDrops R package with default parameters.

#### Normalization

RNA data were normalized using log-normalization with deconvolution library size factors (*106*) and analytic Pearson residuals (*107*). ADT data were normalized using the dsb R package (*108*).

Integrative analysis of RNA and ADT data was performed using weighted nearest neighbor (WNN) algorithm with default parameters (*109*). The resulting WNN graph was used for Louvain clustering and UMAP. Cell annotation was performed manually based on marker gene analysis, following Azimuth’s L1 and L2 ontology levels. Visualization of CITE-seq data was performed using the scCustomViz R package.

DA analysis was performed using the miloR R package (*110*). Milo assigns cells to partially overlapping neighborhoods on the KNN graph and then performs DA testing by modeling cell counts with a generalized linear model (*110*). WNN graphs were used as inputs, with cell assignment to neighborhoods using *k* = 20, prop = 0.2, and a graph refinement scheme. DA testing parameters included fdr.weighting = “graph-overlap,” robust = TRUE, and norm.method = “RLE.” Neighborhoods with spatial FDR < 10% were considered significant. DA neighborhoods were visualized on UMAP embeddings.

### Integrative data analysis

Unsupervised data integration was performed using the MOFA2 R package (*22*, *111*). For PMA and MPL datasets, only cytokine-producing clusters were included; all features were included for other datasets. MOFA models were trained using custom options. Data options: scale_views = TRUE. Model options: num_factors = 9, ard_factors = TRUE, and spikeslab_weights = TRUE. Training options: convergence_mode = “slow,” maxiter = 1000, and drop_factor_threshold = 0.01. MOFA was run for 50 iterations, each with a unique random seed. A model with the highest ELBO was selected for downstream analysis. Missing values in MET and GLY datasets were imputed using the impute function of MOFA2. MDS used the cmdscale function in base R. Principal curve analysis used the princurve R package with default parameters. PERMANOVA was performed using the adonis function in the vegan R package with 1000 permutations.

Supervised data integration was performed using DIABLO in mixOmics R Package (*46*) on the same feature set used for MOFA. The correlation between data blocks was set to 0.4. The number of components and distance metric was tuned using 100 repeats of fivefold cross-validation, suggesting two components and centroid distance. A similar strategy tuned the number of features in each dataset. A permutation significance test based on cross-validation was performed using the DIABLO.test function in the RVAideMemoire R package. Mean and SD of weighted vote accuracy were obtained from 100 repeats of fivefold cross-validation. A consensus plot was generated using the plotIndiv function with block = weighted average. Feature importance was estimated using the block-rank function of the OmicsFold R package with 5000 bootstrap samples.

### Statistical analysis

All statistical analyses were performed in R (v4.2.1) (*112*). Whenever possible, we included age and sex as covariates for statistical adjustment. All data points represent one biological replicate, and technical replicates were not performed. No sample points were omitted from analysis. All testing was two sided. Findings with FDR below 0.1 were considered significant.
