# Supplementary material for: Systems analysis unravels a common rural-urban gradient in immunological profile, function, and metabolic dependencies
Source: Sci Adv. 2025 Apr 30;11(18):eadu0419. doi: 10.1126/sciadv.adu0419 (PMC12042899; doi:10.1126/sciadv.adu0419)
Supplement: Supplementary file 1 — Figs. S1 to S8 Table S1 to S14 [file sciadv.adu0419_sm.pdf]

Science Advances
AAAS

Supplementary Materials for

Systems analysis unravels a common rural-urban gradient in immunological profile, function, and metabolic dependencies

Mikhael D. Manurung *et al.*

Corresponding author: Mikhael D. Manurung, m.d.manurung@lumc.nl;
Maria Yazdanbakhsh, m.yazdanbakhsh@lumc.nl

Sci. Adv. **11**, eadu0419 (2025)
DOI: 10.1126/sciadv.adu0419

This PDF file includes:

Figs. S1 to S8
Table S1 to S14

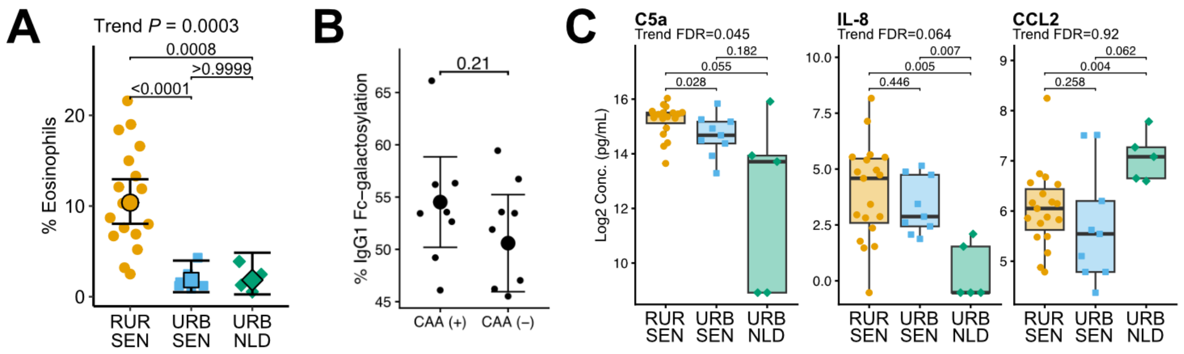

Fig. S1. Eosinophil frequencies and levels of circulating inflammatory markers. Related to Fig. 1.

(A) Eosinophil frequencies in full blood count. Each data point represents an individual sample and error bars indicate adjusted marginal means with 95% confidence intervals. Colors and shapes represent residence categories. Trend test and Tukey-corrected P-values are indicated. **(B)** IgG1 Fc-galactosylation levels in rural Senegalese participants with and without low-intensity schistosomiasis (n=8 and 9, respectively) as determined with CAA assay. Individual data points are shown with larger dots indicating group means. Error bars represent 95% confidence intervals. **(C)** Levels of circulating cytokines as determined using Luminex® assay (n=19 rural Senegalese, 9 urban Senegalese, 5 urban Dutch). Each data point represents an individual sample; boxplots show the median and 1st and 3rd quartiles, with whiskers extending to the maximum/minimum of the respective groups, no further than 1.5X the interquartile range. Jonckheere-Terpstra trend test was used to test for cytokines with graduated changes across the rural-urban gradient (RUR SEN>URB SEN>URB NLD, and vice versa) and the resulting trend-test *P*-values were FDR-corrected. Pairwise comparisons were performed using unpaired Wilcoxon tests. RUR, rural; URB, urban; SEN, Senegal; NLD, the Netherlands; C5a, complement component 5a.

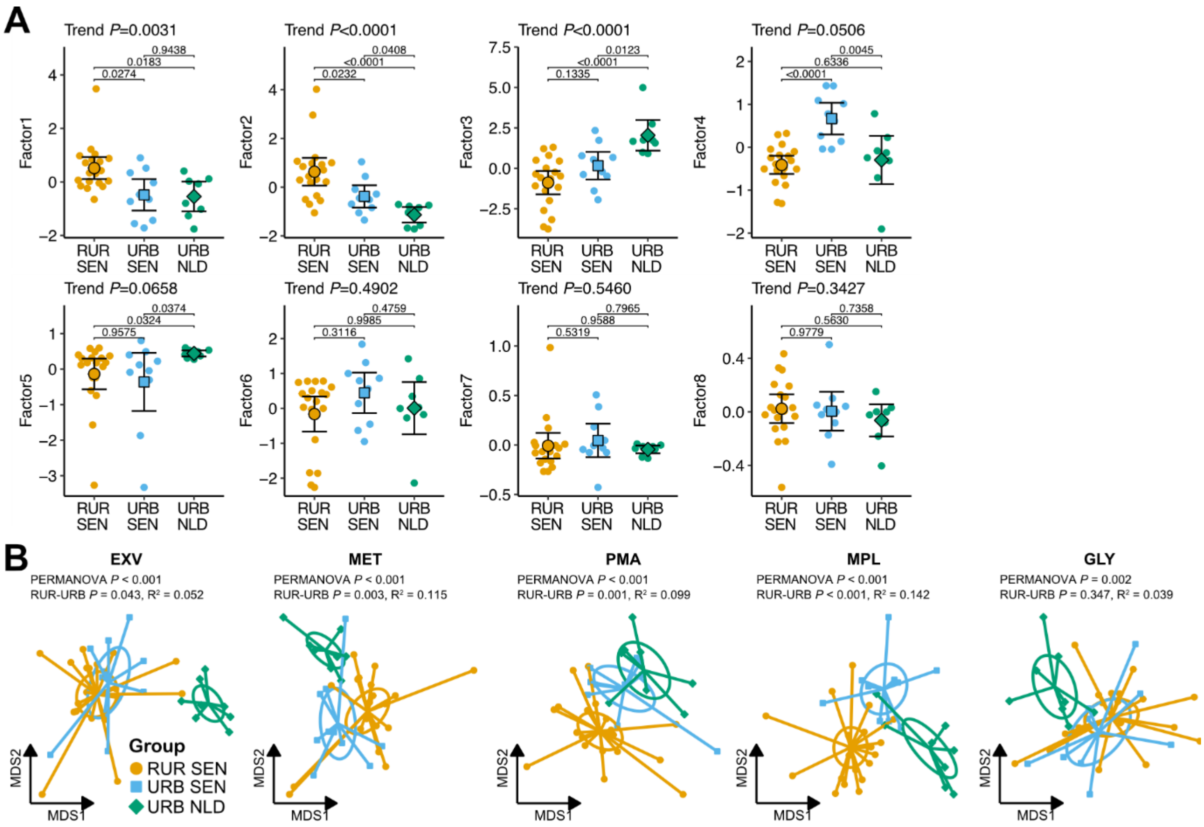

Fig. S2. MOFA factor loadings and sample embeddings. Related to Fig. 2.
(A) MOFA factor loadings. Each data point represents an individual sample and error bars indicate adjusted marginal means with 95% confidence intervals. Colors and shapes indicate residence groups. Linear trend test and Conover post-hoc test for pairwise comparisons *P*-values are indicated. **(B)** Multidimensional scaling (MDS) embedding of samples for each dataset. Points indicate subjects with colors and shapes representing residence groups. Group centroids were compared using PERMANOVA with 1000 permutations; *P*-values of subgroup analyses comparing RUR and URB SEN samples are also shown. MOFA, multi-omics factor analysis; RUR, rural; URB, urban; SEN, Senegal; NLD, the Netherlands; PERMANOVA, permutational analysis of variance.

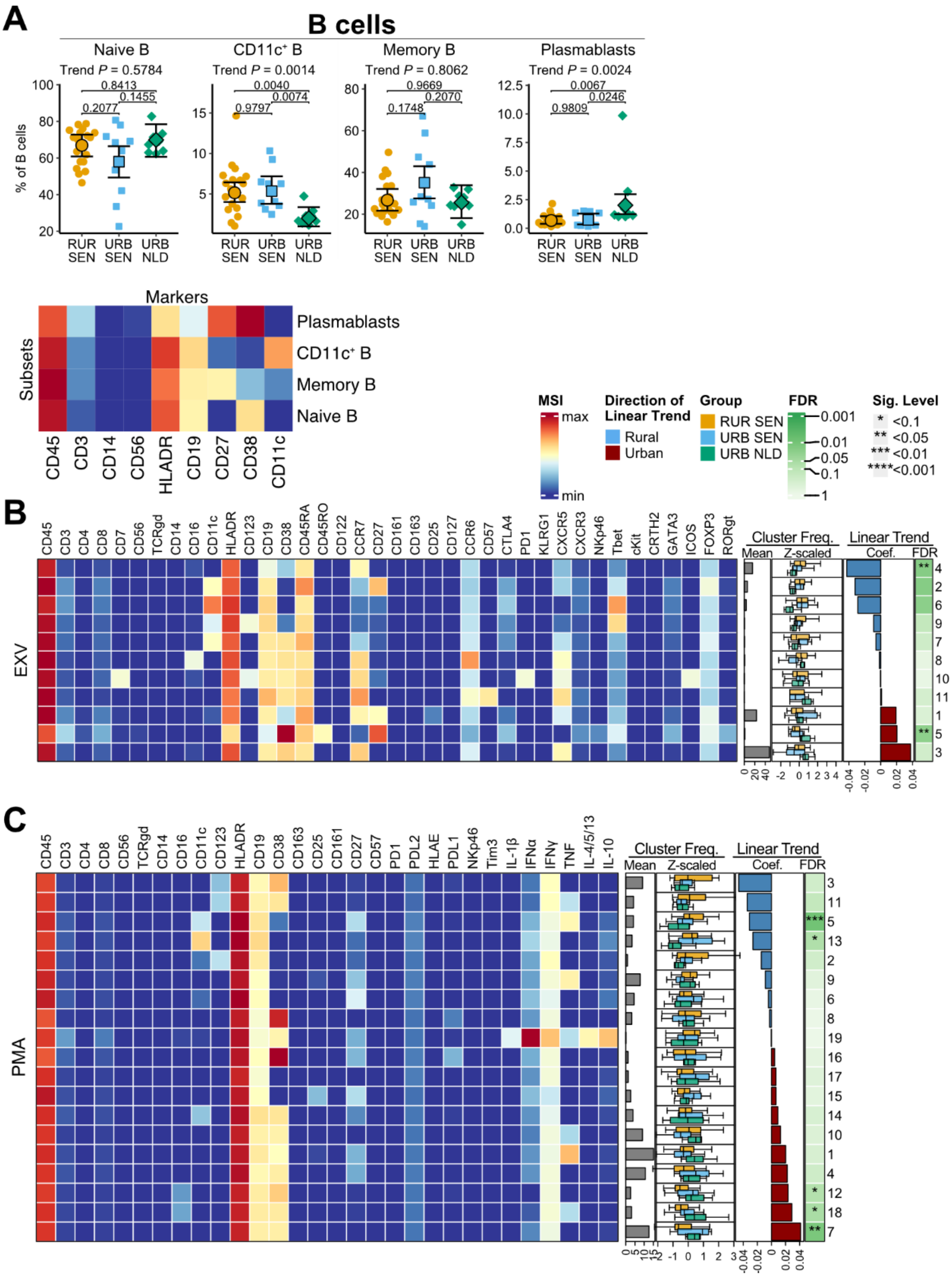

Fig. S3. B cell subsets and clusters. Related to Fig. 3.
(A) *Top*, dot plots showing B cell subset frequencies relative to total B cells in the EXV dataset. Each data point represents an individual sample and error bars indicate adjusted marginal means with 95% confidence intervals. Colors and shapes indicate residence groups. Linear trend test and Tukey-corrected P-values are shown. *Bottom*, heatmap shows the median signal intensity of subsets shown in the top panel. **(B)** Heatmap showing the profiles of 11 B cell clusters in the EXV dataset. Heatmap tile colors indicate median signal intensity. Gray bar plots indicate average cluster frequencies. Boxplots showing z-scaled cluster frequencies; colors indicate residence groups. Red/blue bar plots indicate the direction of the linear trend test coefficient or Spearman’s correlation to the lambda parameter [red,

higher in urban; blue, higher in rural]. Significance levels are indicated. **(C)** Heatmap showing the profiles of 19 B cell clusters in the PMA dataset. Heatmap tile colors indicate median signal intensity. Gray bar plots indicate average cluster frequencies. Boxplots showing z-scaled cluster frequencies; colors indicate residence groups. Red/blue bar plots indicate the direction of the linear trend test coefficient or Spearman's correlation to the lambda parameter [red, higher in urban; blue, higher in rural]. Significance levels are indicated. Grey tile under the Lambda FDR heatmap column indicates non-cytokine^{+} cell clusters that were not included in the MOFA model, hence the absence of correlation between the respective cluster frequencies and the lambda parameter; seven cytokine^{+} cell clusters were included in the MOFA model. RUR, rural; URB, urban; SEN, Senegal; NLD, the Netherlands; EXV, *ex vivo* immunophenotyping.

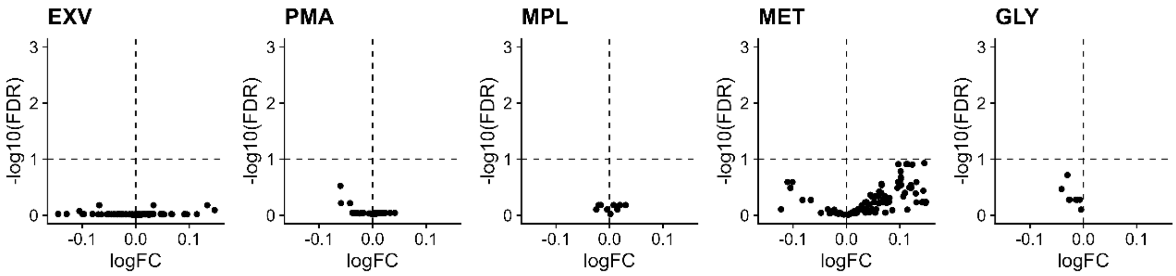

Fig. S4. Comparisons of immune parameters between rural Senegal dwellers with and without low-intensity *Schistosoma* infection.

Volcano plots display differential analysis results comparing immune parameters between participants with (CAA+) and without (CAA-) low-intensity schistosomiasis (n = 8 and 9, respectively). Each point represents an immune parameter. Statistical analysis was performed using the same methodology as the main rural-urban comparisons. Horizontal dashed lines indicate the FDR threshold of 0.1.

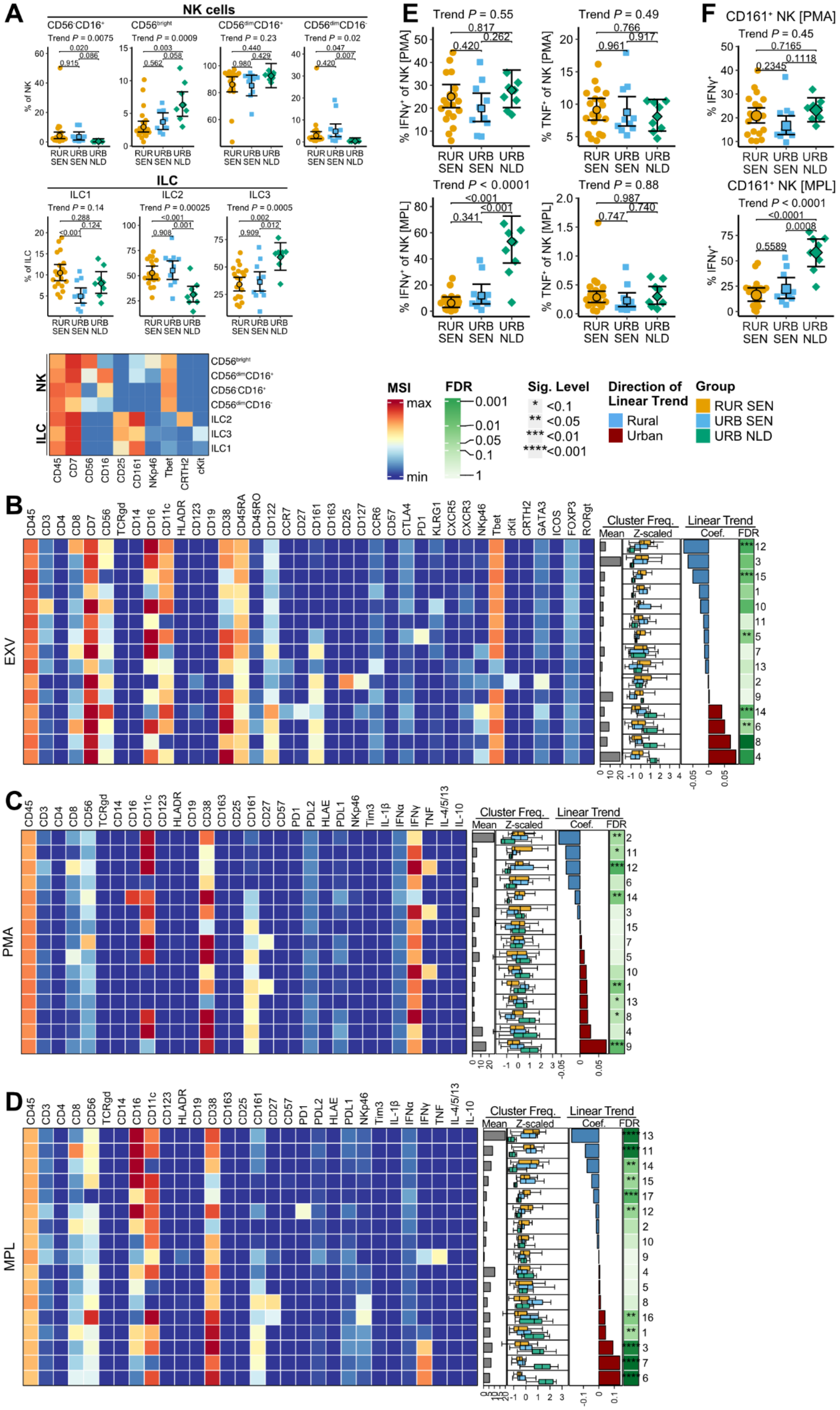
A
NK cells
CD56⁻CD16⁺
Trend P = 0.0075
CD56bright
Trend P = 0.0009
CD56dimCD16⁺
Trend P = 0.23
CD56dimCD16⁻
Trend P = 0.02
% of NK
ILC
ILC1
Trend P = 0.14
ILC2
Trend P = 0.00025
ILC3
Trend P = 0.0005
% of ILC
RUR SEN
URB SEN
URB NLD
NK
ILC
CD56bright
CD56dimCD16⁺
CD56⁻CD16⁺
CD56dimCD16⁻
ILC2
ILC3
ILC1
CD45
CD7
CD56
CD16
CD25
CD161
NKp46
Tbet
CRTH2
cKit
E
Trend P = 0.55
% IFNγ⁺ of NK [PMA]
Trend P = 0.49
% TNF⁺ of NK [PMA]
Trend P < 0.0001
% IFNγ⁺ of NK [MPL]
Trend P = 0.88
% TNF⁺ of NK [MPL]
F
CD161⁺ NK [PMA]
Trend P = 0.45
% IFNγ⁺
CD161⁺ NK [MPL]
Trend P < 0.0001
% IFNγ⁺
MSI
max
min
FDR
0.001
0.01
0.05
0.1
1
Sig. Level
* <0.1
** <0.05
*** <0.01
**** <0.001
Direction of Linear Trend
Rural
Urban
Group
RUR SEN
URB SEN
URB NLD
B
EXV
Cluster Freq.
Mean
Z-scaled
Linear Trend
Coef.
FDR
C
PMA
D
MPL

Fig. S5. NK cell subsets and clusters. Related to Fig. 4.
(A) *Top*, dot plots showing NK cell and ILC subset frequencies relative to total NK cells or ILC respectively. Each data point represents an individual sample and error bars indicate adjusted marginal means with 95% confidence intervals. Colors and shapes indicate residence groups. Linear trend test and Tukey-corrected P-values are shown. *Bottom*, heatmap shows the median signal intensity of subsets shown in the top panel. **(B)** Heatmap showing the profiles of 15 NK clusters in the EXV dataset. Heatmap tile colors indicate median signal intensity. Gray bar plots indicate average cluster frequencies. Boxplots showing z-scaled cluster frequencies; colors indicate residence groups. Red/blue bar plots indicate the direction of the linear trend test coefficient or Spearman's correlation to the lambda parameter [red, higher in urban; blue, higher in rural]. Significance levels are indicated. **(C)** Heatmap showing the profiles of 15 NK cell clusters in the PMA dataset. Heatmap tile colors indicate median signal intensity. Gray bar plots indicate average cluster frequencies. Boxplots showing z-scaled cluster frequencies; colors indicate residence groups. Red/blue bar plots indicate the direction of the linear trend test coefficient or Spearman's correlation to the lambda parameter [red, higher in urban; blue, higher in rural]. Significance levels are indicated. Grey tile under the Lambda FDR heatmap column indicates non-cytokine$^+$ cell clusters that were not included in the MOFA model, hence the absence of correlation between the respective cluster frequencies and the lambda parameter; four cytokine$^+$ cell clusters were included in the MOFA model. **(D)** Heatmap showing the profiles of 17 NK cell clusters in the MPL dataset. Heatmap tile colors indicate median signal intensity. Gray bar plots indicate average cluster frequencies. Boxplots showing z-scaled cluster frequencies; colors indicate residence groups. Red/blue bar plots indicate the direction of the linear trend test coefficient or Spearman's correlation to the lambda parameter [red, higher in urban; blue, higher in rural]. Significance levels are indicated. Grey tile under the Lambda FDR heatmap column indicates non-cytokine$^+$ cell clusters that were not included in the MOFA model, hence the absence of correlation between the respective cluster frequencies and the lambda parameter; four cytokine$^+$ cell clusters were included in the MOFA model. **(E)** Dot plots showing the frequencies of NK cells producing IFNγ or TNF upon PMA/Ionomycin (top) or MPL-A (bottom). Each data point represents an individual sample and error bars indicate adjusted marginal means with 95% confidence intervals. Colors and shapes indicate residence groups. Linear trend test and Tukey-corrected P-values are shown. **(F)** Dot plots showing the frequencies of CD161$^+$NK cells producing IFNγ upon PMA/Ionomycin (top) or MPL-A (bottom). Each data point represents an individual sample and error bars indicate adjusted marginal means with 95% confidence intervals. Colors and shapes indicate residence groups. Linear trend test and Tukey-corrected P-values are shown. RUR, rural; URB, urban; SEN, Senegal; NLD, the Netherlands.

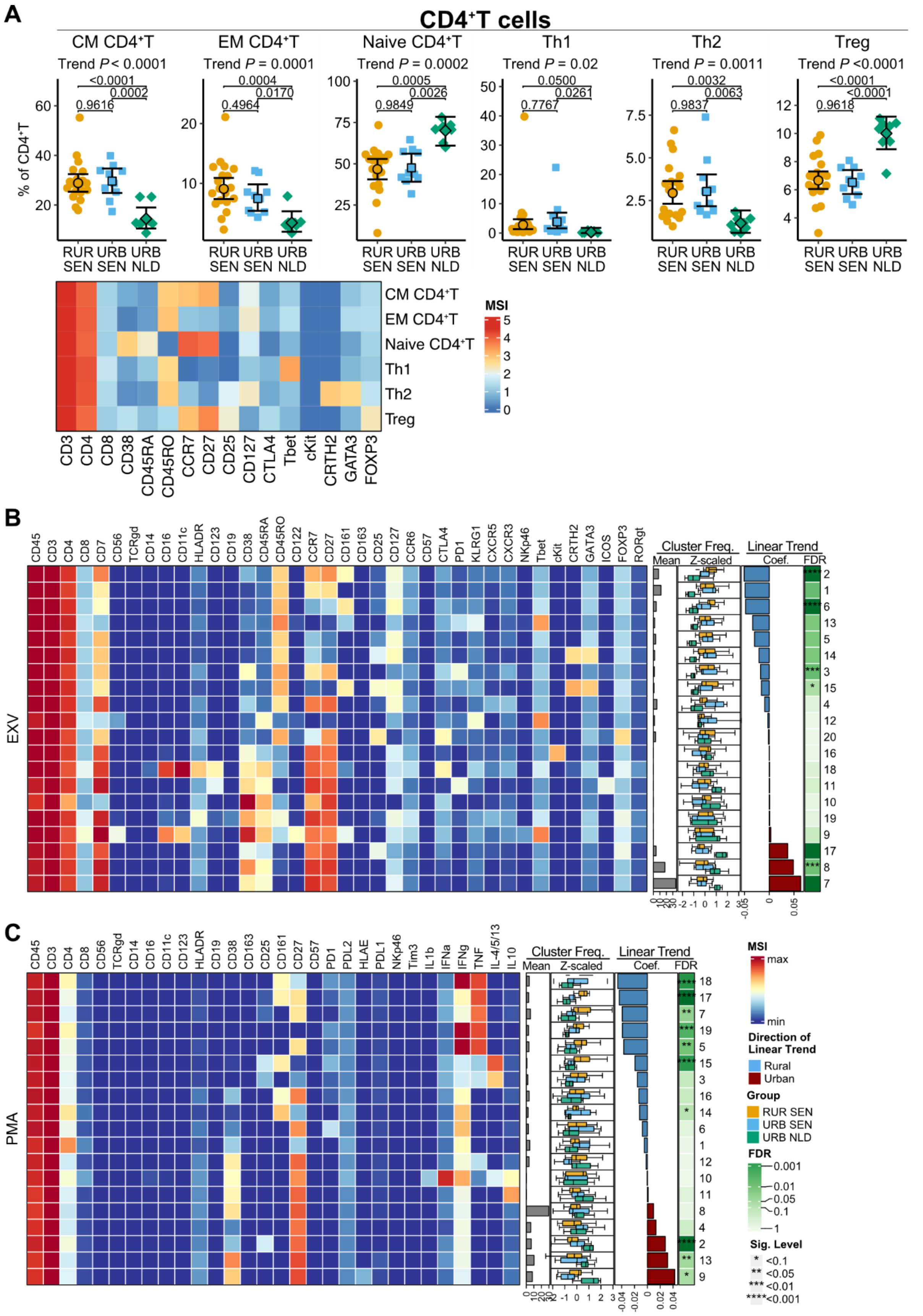

Fig. S6. CD4⁺T cell subsets and clusters. Related to Fig. 5.

(A) *Top*, dot plots showing CD4⁺T cell subset frequencies relative to total CD4⁺T cells in EXV dataset. Each data point represents an individual sample and error bars indicate adjusted

marginal means with 95% confidence intervals. Colors and shapes indicate residence groups. Linear trend test and Tukey-corrected *P*-values are shown. *Bottom*, heatmap shows the median signal intensity of subsets shown in the top panel. **(B)** Heatmap showing the profiles of 20 $CD4^+$T cell clusters in the EXV dataset. Heatmap tile colors indicate median signal intensity. Gray bar plots indicate average cluster frequencies. Boxplots showing z-scaled cluster frequencies; colors indicate residence groups. Red/blue bar plots indicate the direction of the linear trend test coefficient or Spearman's correlation to the lambda parameter [red, higher in urban; blue, higher in rural]. Significance levels are indicated. **(C)** Heatmap showing the profiles of 19 $CD4^+$T cell clusters in the PMA dataset. Heatmap tile colors indicate median signal intensity. Gray bar plots indicate average cluster frequencies. Boxplots showing z-scaled cluster frequencies; colors indicate residence groups. Red/blue bar plots indicate the direction of the linear trend test coefficient or Spearman's correlation to the lambda parameter [red, higher in urban; blue, higher in rural]. Significance levels are indicated. Grey tile under the Lambda FDR heatmap column indicates non-cytokine$^+$ cell clusters that were not included in the MOFA model, hence the absence of correlation between the respective cluster frequencies and the lambda parameter; 10 cytokine$^+$ cell clusters were included in the MOFA model.

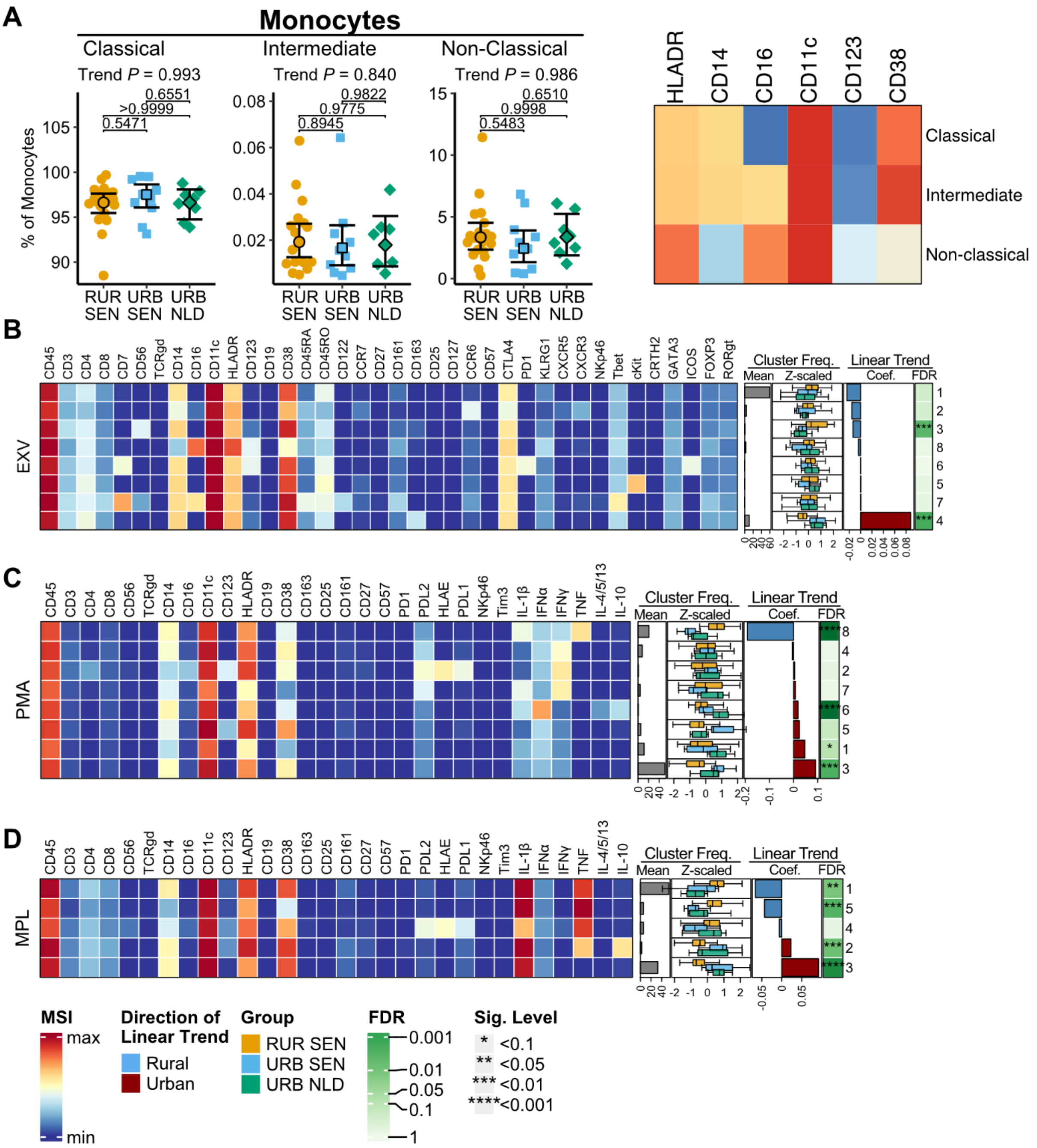

Fig. S7. Monocyte subsets and clusters. Related to Fig. 6.
(A) Left, Boxplots showing monocyte subset frequencies relative to parent lineage. Each data point represents an individual sample and error bars indicate adjusted marginal means with 95% confidence intervals. Colors and shapes indicate residence groups. Linear trend test and Tukey-corrected P-values are shown. Right, heatmap showing the median signal intensity of subsets is shown in the left panel. **(B)** Heatmap showing the profiles of 8 monocyte clusters in the EXV dataset. Heatmap tile colors indicate median signal intensity. Gray bar plots indicate average cluster frequencies. Boxplots showing z-scaled cluster frequencies; colors indicate residence groups. Red/blue bar plots indicate the direction of the linear trend test coefficient or Spearman's correlation to the lambda parameter [red, higher in urban; blue, higher in rural]. Significance levels are indicated. **(C)** Heatmap showing the profiles of 8 monocyte clusters in the PMA dataset. Heatmap tile colors indicate median signal intensity. Gray bar plots indicate average cluster frequencies. Boxplots showing z-scaled cluster

frequencies; colors indicate residence groups. Red/blue bar plots indicate the direction of the linear trend test coefficient or Spearman's correlation to the lambda parameter [red, higher in urban; blue, higher in rural]. Significance levels are indicated. Grey tile under the Lambda FDR heatmap column indicates non-cytokine^{+} cell clusters that were not included in the MOFA model, hence the absence of correlation between the respective cluster frequencies and the lambda parameter; four cytokine^{+} cell clusters were included in the MOFA model. **(D)** Heatmap showing the profiles of 5 monocyte clusters in the MPL dataset. Heatmap tile colors indicate median signal intensity. Gray bar plots indicate average cluster frequencies. Boxplots showing z-scaled cluster frequencies; colors indicate residence groups. Red/blue bar plots indicate the direction of the linear trend test coefficient or Spearman's correlation to the lambda parameter [red, higher in urban; blue, higher in rural]. Significance levels are indicated. Grey tile under the Lambda FDR heatmap column indicates non-cytokine^{+} cell clusters that were not included in the MOFA model, hence the absence of correlation between the respective cluster frequencies and the lambda parameter; five cytokine^{+} cell clusters were included in the MOFA model.

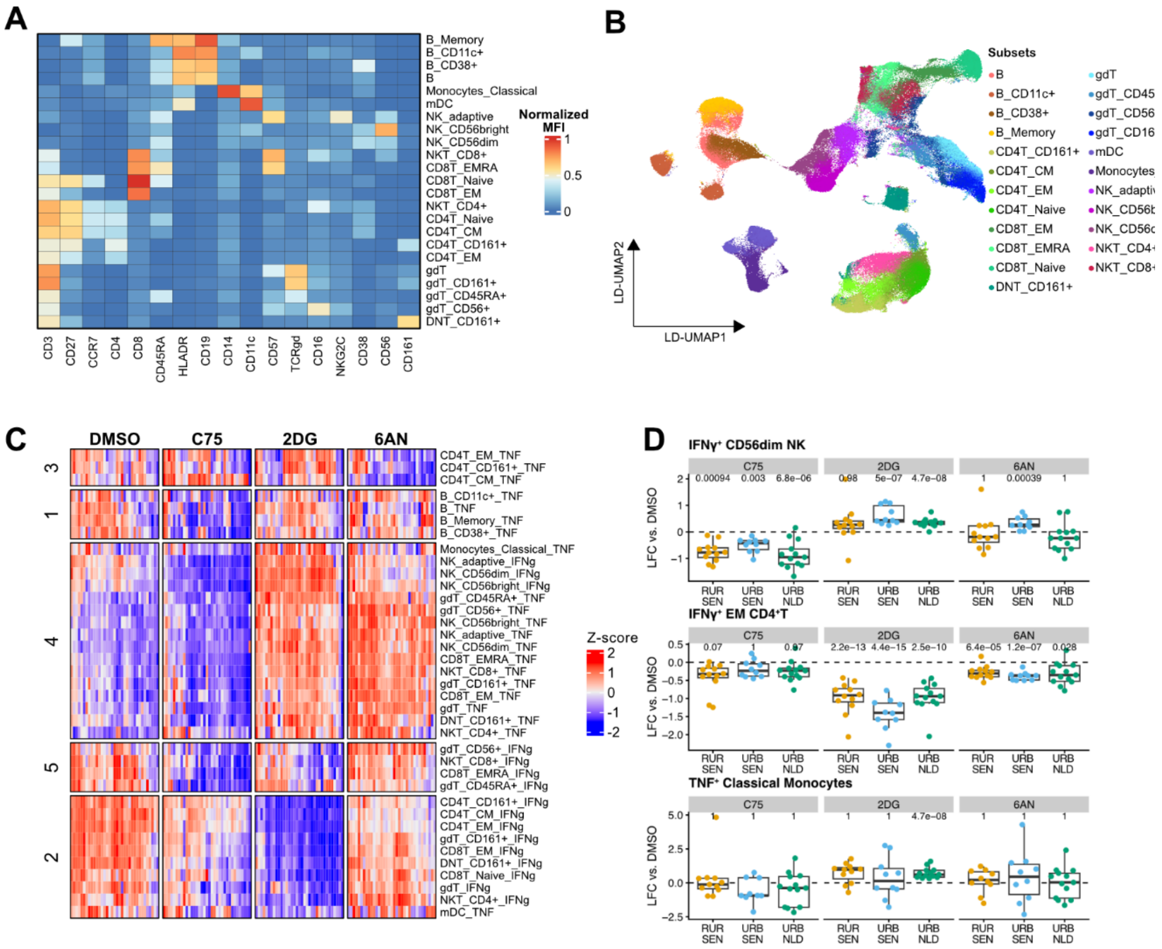

Fig. S8. Metabolic enzyme inhibition analysis. Related to Fig. 8.
(A) Heatmap showing median fluorescence intensity of surface markers of 23 immune cell subsets identified using flow cytometry. **(B)** LD-UMAP embedding of immune cell subsets shown in panel A. Cells were downsampled to 10,000 cells for each of the 23 cell subsets. **(C)** Heatmap showing z-scores of iMFI across individuals and pre-treatment conditions. Columns are samples, and rows are distinct cytokine-subset pairs. Rows are split according to the clustering of features, with consensus clustering to determine the number of clusters. Color of heatmap tiles represents within-subject z-scored iMFI. **(D)** Representative boxplots of $\log_2$ fold-change (LFC) in cytokine response in each pre-treatment condition over DMSO control. Colors represent the group. FDR-corrected p-values shown above boxplots were obtained from linear mixed models comparing iMFIs upon metabolic enzyme inhibition over DMSO for each group.

Table S1. Patient Demographics.

		Residence			
Characteristic	Overall, N = 37[1]	Rural Senegal N = 19[1]	Urban Senegal N = 10[1]	Urban Netherlands N = 8[1]	*P*-value[2]
Age, years	26.0 (18.0 - 37.0)	26.0 (18.0 - 36.0)	27.0 (21.0 - 32.0)	25.0 (20.0 - 37.0)	0.9
Sex, female	21 (57%)	12 (63%)	4 (40%)	5 (63%)	0.5
Height, kg	1.71 (0.08)	1.68 (0.08)	1.74 (0.11)	1.71 (0.04)	0.3
Missing	10	8	0	2	
Weight, kg	67 (13)	65 (13)	68 (13)	73 (16)	0.6
Missing	6	4	0	2	
BMI, kg/m²	22.9 (3.9)	22.2 (3.8)	22.4 (3.3)	24.8 (4.8)	0.6
Missing	10	8	0	2	
Systolic blood pressure, mmHg	115 (100, 120)	120 (100, 120)	110 (100, 110)	124 (114, 136)	0.064
Missing	4	4	0	0	
Diastolic blood pressure, mmHg	78 (70, 80)	70 (60, 80)	80 (73, 80)	77 (71, 87)	0.3
Missing	4	4	0	0	
Random blood glucose, mMol/L	4.80 (4.40, 5.20)	5.10 (4.30, 5.20)	4.75 (4.53, 4.98)	4.60 (4.48, 4.88)	0.6
Current or past tobacco use	3 (8.1%)	1 (5.3%)	1 (10%)	1 (13%)	0.8
Passive smoking	14 (38%)	5 (26%)	4 (40%)	5 (63%)	0.2
CAA[3]	8 (21%)	8/17 (47%)	0 (0%)	0 (0%)	0.016
Missing	6	2	0	4	

[1]Median (Range); n (%); Mean (SD); Median (IQR)
[2]Kruskal-Wallis rank sum test; Fisher's exact test
[3] Circulating Anodic Antigen for detection of Schistosomiasis.

Table S2. Lifestyle and socioeconomic factors.

Characteristic	Residence: Rural Senegal, N = 19[1]	Urban Senegal, N = 10[1]	Urban Netherlands, N = 8[1]	*P*-value[2]
Lived in current residence ≥5 years	19 (100%)	10 (100%)	4 (50%)	0.001
Years spent in current residence category, normalized by age	0.99 (0.87)	1.0 (1.0)	1.0 (1.0)	0.6
Household size	14 (8)	8 (5)	3 (1)	<0.001
Missing	3	1	0	
Floor coverings				<0.001
No	14 (82%)	0 (0%)	0 (0%)	
Partial	1 (5.9%)	2 (20%)	0 (0%)	
Yes	2 (12%)	8 (80%)	8 (100%)	
Missing	2	0	0	
Wall coverings				<0.001
No	13 (76%)	0 (0%)	0 (0%)	
Partial	1 (5.9%)	0 (0%)	0 (0%)	
Yes	3 (18%)	10 (100%)	8 (100%)	
Missing	2	0	0	
Contact with animals	16 (94%)	6 (60%)	6 (75%)	0.080
Missing	2	0	0	
Contact with animal excrement	12 (63%)	4 (40%)	2 (25%)	0.14

[1]n (%); Mean (Minimum); Mean (SD)

[2]Fisher's exact test; Kruskal-Wallis rank sum test

Table S3. Differential analysis statistics for circulating cytokines assay.

	Group mean (log2)				Wilcoxon test P-value		
Analyte	RUR SEN	URB SEN	URB NLD	trend_test_FDR	RUR SEN vs URB SEN	RUR SEN vs URB NLD	URB SEN vs URB NLD
Complement component 5a (C5a)	15,224	14,662	12,278	0,045	0,028	0,055	0,182
IL-8/CXCL8	3,960	3,342	0,407	0,064	0,446	0,005	0,007
CXCL10/IP-10	5,494	4,887	4,935	0,28	0,068	0,088	0,797
CD40L/TNFSF5	9,887	9,516	9,129	0,306	0,41	0,063	0,147
IL-18	6,821	6,515	6,559	0,575	0,168	0,394	0,699
HGF	5,969	6,117	5,317	0,772	0,676	0,064	0,009
CCL24/Eotaxin-2	7,620	7,632	8,568	0,921	0,699	0,088	0,317
VEGF	3,757	4,024	3,195	0,921	0,337	0,102	0,071
CCL2/MCP-1	6,040	5,715	7,076	0,921	0,258	0,004	0,062

Jonckheere-Terpstra trend test was used to test for cytokines with graduated changes across the rural-urban gradient (RUR SEN>URB SEN>URB NLD, and vice versa) and the resulting trend-test *P*-values were FDR-corrected with the Benjamini-Hochberg method. Cells highlighted in green indicate statistically significant results.

Table S4. Overview of data generated in the study.

		Cohort 1			Cohort 2			Cohort 3		
Instrument	Assays	RUR SEN	URB SEN	URB NLD	RUR IDN	URB IDN	URB NLD	RUR SEN	URB SEN	URB NLD
Mass Cytometry	Ex vivo phenotyping	19	10	8	8	8	8			
	PMA/Ionomycin stimulation	19	10	8	8	8	8			
	MPL stimulation	19	10	8						
Spectral Flow	Met-Flow	14	9	6						
	Metabolic enzyme inhibition + ICCS							12	10	13
Mass Spectrometry	IgG glycosylation	18	7	4						
Multiplex immunoassay	Heparin plasma	19	9	5						
CITE-Seq	Ex vivo	2	2	2						

ICCS, intracellular cytokine staining; RUR SEN, rural Senegal; URB SEN, urban Senegal; URB NLD, urban Netherlands; RUR IDN, rural Indonesia; URB IDN, urban Indonesia.

Cells highlighted in gray indicate no assays were performed.

Table S5. Differential abundance analysis statistics for B cell clusters.

dataset	lineage	cluster	annotation	linear_trend_coef	linear_trend_fdr	quadratic_trend_fdr	significant_linear	cytokine_expressing
EXV	B	EXV.4	B	-0.0426	0.0176	0.5978	**	N/A
EXV	B	EXV.2	B_Memory_CD11c^{+}	-0.0323	0.0176	0.081		N/A
EXV	B	EXV.6	B_CD11c^{+}_CTLA4^{+}Tbet^{+}	-0.0287	0.0218	0.0083		N/A
EXV	B	EXV.9	B_CD11c^{+}_CCR6^{+}Tbet^{+}	-0.0089	0.1541	0.2284		N/A
EXV	B	EXV.7	B_CD11c^{+}_CCR6^{+}	-0.006	0.2501	0.3557		N/A
EXV	B	EXV.8	B_CD16^{+}CCR6^{+}	-0.0016	0.7461	0.0026		N/A
EXV	B	EXV.10	B_CCR6^{+}PD1^{+}CXCR5^{+}ICOS^{+}	-3,00E-04	0.7963	0.9028		N/A
EXV	B	EXV.11	B_CCR6^{+}CD57^{+}	0.0011	0.3151	0.4366		N/A
EXV	B	EXV.1	B_Memory	0.0192	0.4274	0.2018		N/A
EXV	B	EXV.5	B_Plasmablasts	0.0204	0.0232	0.2256	**	N/A
EXV	B	EXV.3	B_CCR6^{+}	0.0376	0.2745	0.0162		N/A
PMA	B	PMA.3	B_CD123^{+}CD38^{+}	-0.0464	0.268	0.7015		negative
PMA	B	PMA.11	B_CD123^{+}CD38^{+}TNF^{+}	-0.0344	0.1569	0.1916		positive
PMA	B	PMA.5	B_CD11c^{+}CD27^{+}TNF^{+}	-0.0313	0.0091	0.8707	***	positive
PMA	B	PMA.13	B_CD11c^{+}	-0.0262	0.0707	0.0445	*	negative
PMA	B	PMA.2	B_CD123^{+}	-0.0144	0.268	0.632		negative
PMA	B	PMA.9	B_CD27^{+}TNF^{+}	-0.0087	0.5749	0.0538		positive

PMA	B	PMA.6	B_CD27^{+}	-0.0042	0.7967	0.0747		negative
PMA	B	PMA.8	B_CD38^{hi}	-0.0026	0.9181	0.4412		negative
PMA	B	PMA.19	B_CD27^{+}CD38^{+}IL10^{+}IFNα^{+}TNF^{+}	-2,00E-04	0.9483	0.4979		positive
PMA	B	PMA.16	B_CD27^{+}CD38^{hi}	0.0045	0.6493	0.9476		negative
PMA	B	PMA.17	B	0.0064	0.4988	0.0776		negative
PMA	B	PMA.15	B_CD25^{+}CD27^{+}	0.0066	0.615	0.0334		negative
PMA	B	PMA.14	B_CD11c^{+}CD38^{+}	0.0092	0.374	0.023		negative
PMA	B	PMA.10	B_CD38^{+}TNF^{+}	0.0129	0.4558	0.0195		positive
PMA	B	PMA.1	B_CD27^{+}CD38^{+}TNF^{+}	0.0202	0.3656	0.0352		positive
PMA	B	PMA.4	B_CD27^{+}CD38^{+}	0.0224	0.245	0.1794		negative
PMA	B	PMA.12	B_CD16^{+}CD38^{+}	0.0236	0.0806	0.417	*	negative
PMA	B	PMA.18	B_CD16^{+}CD38^{+}TNF^{+}	0.0289	0.0584	0.5979	*	positive
PMA	B	PMA.7	B_CD38^{+}	0.0414	0.0126	0.0099	**	negative

Rows of linear_trend_fdr highlighted in green indicate statistically significant results. *FDR<0.1, **<0.05, ***<0.01, ****<0.001.

Table S6. Differential analysis results for comparisons of immune parameters between rural Senegal dwellers with and without low-intensity *Schistosoma* infection by CAA.

dataset	feature	logFC	t	P.Value	adj.P.Val
EXV	B	0,134	2,95	0,0107	0,662
EXV	T_H2 CD161$^+$	0,0332	2,71	0,0169	0,662
EXV	γδT DN	-0,0683	-2,67	0,0186	0,662
EXV	CD8T EMRA	0,148	2,41	0,0303	0,81
EXV	T_H1 PD1$^+$	-0,105	-2,28	0,0393	0,84
EXV	mDC CXCR3$^+$	0,0263	2,01	0,0638	0,952
EXV	B CCR6$^+$	-0,0995	-1,91	0,0775	0,952
EXV	B CD11c$^+$CCR6$^+$	0,0229	1,86	0,084	0,952
EXV	CD8T Naive	-0,145	-1,72	0,108	0,952
EXV	B CD16$^+$CCR6$^+$	0,0157	1,7	0,111	0,952
PMA	B CD27$^+$CD38$^+$TNF$^+$	-0,0602	-2,95	0,0103	0,299
PMA	B CD27$^+$TNF$^+$	-0,0421	-2,03	0,0619	0,61
PMA	CD8T_CD161$^+$IFNγ$^+$TNF$^+$	-0,059	-2,01	0,0631	0,61
PMA	CD4T_CD161$^+$IFNγ$^+$TNF^{hi}	-0,035	-1,4	0,183	0,909
PMA	NK_CD11c$^+$CD38$^+$CD161$^+$IFNγ$^+$	-0,0329	-1,25	0,233	0,909
PMA	CD4T_CD38$^+$CD27$^+$TNF$^+$	0,0192	1,22	0,241	0,909
PMA	Monocytes_CD16$^+$_CD123^{hi}CD38^{hi}PDL2$^+$HLAE$^+$PDL1$^+$	-0,0208	-1,05	0,312	0,909
PMA	NK_CD11c$^+$_CD38$^+$CD161$^+$IFNγ$^+$TNF$^+$	0,0416	1,02	0,324	0,909

PMA	CD4T_CD25^{+}_TNF^{+}IL4IL5IL13^{+}	0,0139	1,02	0,325	0,909
PMA	B_CD27^{+}_CD38^{+}IL10^{+}IFNα^{+}TNF^{+}	0,00515	0,92	0,373	0,909
MPL	Monocytes_CD38^{+}_CD14^{+}TNF^{+}IL10^{+}IL1β^{+}	-0,0201	-1,36	0,194	0,659
MPL	NK_CD56^{dim}CD16$^{–}$_CD38^{+}CD11c^{+}IFNγ^{+}TNF^{+}	0,0198	1,32	0,206	0,659
MPL	B_IFNγ^{+}_TNF^{+}IL10^{+}	0,00777	1,19	0,254	0,659
MPL	B_memory_TNF^{+}	-0,0162	-1,08	0,298	0,659
MPL	NK_CD56^{dim}CD16-_CD8^{+}CD38^{+}CD11c^{+}IFNγ^{+}	0,019	0,989	0,339	0,659
MPL	Monocytes_CD14^{+}TNF^{+}IL1β^{+}	0,0302	0,946	0,359	0,659
MPL	Monocytes_CD38^{+}CD14^{+}TNF^{+}IL1β^{+}	-0,0245	-0,563	0,582	0,791
MPL	B_Plasmablasts_CD38^{+}PDL1^{+}	-0,00462	-0,494	0,628	0,791
MPL	NK_CD56^{dim}CD16$^{–}$_CD38^{+}IFNγ^{+}	0,0145	0,449	0,66	0,791
MPL	NK_CD56^{dim}CD16^{dim}_CD38^{+}CD11c^{+}IFNγ^{+}	-0,0249	-0,367	0,719	0,791
MET	CD4T__Naive.ACC1.p80	0,146	4,36	0,0015	0,118
MET	Monocytes__Intermediate.Cytc.p80	-0,261	-5,49	0,0017	0,118
MET	CD4T__CD45RO$^{–}$.ACC1.p80	0,153	4,26	0,00175	0,118
MET	CD4T__CD45RO^{+}.PKM.p80	0,114	4,04	0,00245	0,123
MET	CD4T__EM.PKM.p80	0,0971	3,94	0,00289	0,123
MET	CD4T__CM.PKM.p80	0,112	3,91	0,00303	0,123
MET	Bcells.PKM.p80	0,123	3,78	0,00372	0,125
MET	Monocytes__Intermediate.CPT1A.p80	-0,546	-4,46	0,00506	0,142

MET	CD4T__T_H2.ACC1.p80	0,238	3,39	0,0071	0,165
MET	CD4T__CD161$^+$.PKM.p80	0,101	3,37	0,00737	0,165
GLY	IgG1_Sialylation	-0,0298	-2,52	0,024	0,192
GLY	IgG1_Galactosylation	-0,0407	-1,84	0,0856	0,342
GLY	IgG1_Fucosylation	-0,0255	-1,29	0,216	0,529
GLY	IgG2_Galactosylation	-0,027	-0,917	0,375	0,529
GLY	IgG2_Fucosylation	-0,00653	-0,835	0,418	0,529
GLY	IgG2_Bisection	-0,011	-0,812	0,431	0,529
GLY	IgG2_Sialylation	-0,0139	-0,756	0,463	0,529
GLY	IgG1_Bisection	-0,00429	-0,265	0,795	0,795

p80, 80th percentile; DN, double negative.

Statistical analysis was performed using limma R package, following the procedure for the main rural-urban comparisons. *P*-values were corrected for multiple testing using Benjamini-Hochberg FDR correction method per each datasets with FDR threshold set at 0.1. For each dataset, only top 10 results are shown.

Table S7. Differential abundance analysis statistics for NK cell clusters.

dataset	lineage	cluster	annotation	linear_trend_coef	linear_trend_fdr	quadratic_trend_fdr	significant_linear	cytokine_expressing
EXV	NKILC	EXV.12	NK_CD56^{dim}CD16^{+}_CD8^{+}CD38^{+}	-0.0787	0.0019	0.4891	***	N/A
EXV	NKILC	EXV.3	NK_CD56^{dim}CD16^{+}_CD38^{+}	-0.0652	0.0083	0.0761		N/A
EXV	NKILC	EXV.15	NK_CD56$^{–}$CD16^{+}	-0.05	0.0055	0.1788	***	N/A
EXV	NKILC	EXV.1	NK_CD56^{dim}CD16$^{–}$	-0.0294	0.031	0.0132		N/A
EXV	NKILC	EXV.10	NK_CD56^{dim}CD16^{+}_KLRG1^{+}	-0.0257	0.0037	0.0289		N/A
EXV	NKILC	EXV.11	NK_CD56^{dim}CD16^{+}	-0.0159	0.4275	0.1224		N/A
EXV	NKILC	EXV.5	NK_CD56^{dim}CD16^{+}_CD38^{+}PD1^{+}	-0.014	0.0186	0.6452	**	N/A
EXV	NKILC	EXV.7	NK_CD56^{dim}CD16^{+}_CD8^{+}CD38^{+}CD161^{+}	-0.0131	0.752	0.6112		N/A
EXV	NKILC	EXV.13	NK_CD56^{dim}CD16^{+}_CCR6^{+}	-0.0099	0.2109	0.5213		N/A
EXV	NKILC	EXV.2	NK_CD56^{dim}CD16$^{–}$_CD161^{+}CD25^{+}cKit^{+}	-0.0016	0.4488	0.6385		N/A
EXV	NKILC	EXV.9	NK_CD56^{dim}CD16^{+}_CD38^{+}CD161^{+}	0.002	0.915	0.0289		N/A
EXV	NKILC	EXV.14	NK_CD56^{bright}_CD38^{+}NKp46^{+}	0.0412	0.0036	0.6307	***	N/A
EXV	NKILC	EXV.6	NK_CD56^{dim}CD16^{+}_ CD8^{+}CD38^{+}CD161^{+}NKp46^{+}	0.0508	0.0442	0.1224	**	N/A
EXV	NKILC	EXV.8	NK_CD56^{dim}CD16^{+}_CD161^{+}	0.0688	1,00E-04	0.0812		N/A
EXV	NKILC	EXV.4	NK_CD56dimCD16^{+}_ CD38^{+}CD161^{+}NKp46^{+}	0.0862	6,00E-04	0.0037		N/A
PMA	NK	PMA.2	NK_CD11c^{+}CD38^{+}	-0.0564	0.0469	0.1414		negative

PMA	NK	PMA.11	NK_CD11c$^+$	-0.0379	0.0604	0.742		negative
PMA	NK	PMA.12	NK_CD8$^+$IFNγ+TNF$^+$	-0.0369	0.0024	0.2165	***	positive
PMA	NK	PMA.6	NK_CD38$^+$	-0.0306	0.2266	0.3537		negative
PMA	NK	PMA.14	NK_CD16$^+$CD11c$^+$CD38$^+$CD161$^+$	-0.0131	0.0132	0.3229		negative
PMA	NK	PMA.3	NK_CD11c$^+$CD38$^+$CD161$^+$IFNγ$^+$TNF$^+$	-0.0064	0.7083	0.2916		positive
PMA	NK	PMA.15	NK_CD11c$^+$CD161$^+$	0.0014	0.9505	0.3619		negative
PMA	NK	PMA.7	NK_CD11c$^+$CD38$^+$CD161$^+$CD27$^+$	0.0042	0.7967	0.4003		negative
PMA	NK	PMA.5	NK_CD8$^+$CD11c$^+$CD38$^+$CD161$^+$	0.0117	0.6846	0.7841		negative
PMA	NK	PMA.10	NK_CD38$^+$CD161$^+$IFNγ$^+$TNF$^+$	0.017	0.1081	0.9855		positive
PMA	NK	PMA.1	NK_CD38$^+$CD161$^+$CD27$^+$	0.0185	0.0137	0.032		negative
PMA	NK	PMA.13	NK_CD161$^+$	0.0198	0.0959	0.9399		negative
PMA	NK	PMA.8	NK_CD38$^+$CD161$^+$IFNγ$^+$	0.0206	0.0959	0.0139	*	positive
PMA	NK	PMA.4	NK_CD11c$^+$CD38$^+$CD161$^+$	0.0284	0.3399	0.7728		negative
PMA	NK	PMA.9	NK_CD38$^+$CD161$^+$	0.0702	0.0045	0.4003		negative
MPL	NK	MPL.13	NK_CD56^{dim}CD16$^+$_CD38$^+$CD11c$^+$	-0.17662	0.000156	0.11009		negative
MPL	NK	MPL.11	NK_CD56^{dim}CD16$^+$_CD8$^+$CD38$^+$CD11c$^+$	-0.08753	0.00015	0.00504		negative
MPL	NK	MPL.14	NK_CD56^{dim}CD16$^+$_CD38$^+$	-0.07644	0.015108	0.23601		negative
MPL	NK	MPL.15	NK_CD56^{dim}CD16$^+$_CD11c$^+$	-0.04649	0.036011	0.13577		negative
MPL	NK	MPL.17	NK_CD56$^-$CD16^{dim}_CD11c$^+$	-0.03402	0.002976	0.69834		negative
MPL	NK	MPL.12	NK_CD56^{dim}CD16$^+$_CD38$^+$CD11c$^+$PD1$^+$	-0.01807	0.030774	0.94867		negative

MPL	NK	MPL.2	NK_CD56^{dim}CD16^{dim}_CD8^{+}CD11c^{+}	-0.01617	0.101117	0.72725		negative
MPL	NK	MPL.10	NK_CD56^{dim}CD16^{-}_CD38^{+}CD11c^{+}	-0.00972	0.294918	0.79952		negative
MPL	NK	MPL.9	NK_CD56^{dim}CD16^{-}_CD38^{+}CD11c^{+}IFNγ^{+}TNF^{+}	-0.00031	1.0	0.49521		positive
MPL	NK	MPL.4	NK_CD56^{dim}CD16^{dim}_CD38^{+}CD11c^{+}	0.00616	0.858232	0.69256		negative
MPL	NK	MPL.5	NK_CD56^{dim}CD16^{dim}_CD38^{+}	0.00654	0.798235	0.58736		negative
MPL	NK	MPL.8	NK_CD56^{dim}CD16^{-}_CD38^{+}CD27^{+}NKp46^{+}	0.01133	0.423502	0.05786		negative
MPL	NK	MPL.16	NK_CD56^{bright}_CD38^{+}CD11c^{+}CD27^{+}NKp46^{+}	0.0401	0.017088	0.53403		negative
MPL	NK	MPL.1	NK_CD56^{dim}CD16^{dim}_CD8^{+}CD38^{+}CD11c^{+}	0.0438	0.048897	0.61703		negative
MPL	NK	MPL.3	NK_CD56^{dim}CD16^{dim}_CD38^{+}CD11c^{+}IFNγ^{+}	0.09208	0.000263	0.56919	***	positive
MPL	NK	MPL.7	NK_CD56^{dim}CD16^{-}_CD38^{+}IFNγ^{+}	0.13637	0	0.1067	***	positive
MPL	NK	MPL.6	NK_CD56^{dim}CD16^{-}_CD8^{+}CD38^{+}CD11c^{+}IFNγ^{+}	0.13758	0	0.69384	***	positive

Rows of linear_trend_fdr highlighted in green indicate statistically significant results. *FDR<0.1, **<0.05, ***<0.01, ****<0.001.

Table S8. Differential abundance analysis statistics for $CD4^+$T cell clusters.

dataset	lineage	cluster	annotation	linear_ trend_coef	linear_ trend_fdr	quadratic _trend_fdr	significant _linear	cytokine _expressing
EXV	CD4T	EXV.2	CD4T_CM_$CD161^+$	-0.05066	2,00E-05	0.82371	***	N/A
EXV	CD4T	EXV.1	CD4T_CM	-0.04898	0.00577	0.02885		N/A
EXV	CD4T	EXV.6	CD4T_EM_$CD161^+$	-0.04778	2,00E-05	0.93723	***	N/A
EXV	CD4T	EXV.13	T_H1_$PD1^+$	-0.03346	0.01249	0.04901		N/A
EXV	CD4T	EXV.5	CD4T_EM	-0.02971	0.01249	0.08118		N/A
EXV	CD4T	EXV.14	T_H2	-0.02079	0.01249	0.08118		N/A
EXV	CD4T	EXV.3	CD4T_CM_$CTLA4^+PD1^+$	-0.01756	0.00577	0.1788	***	N/A
EXV	CD4T	EXV.15	T_H2_$CD161^+$	-0.01591	0.06643	0.72186	*	N/A
EXV	CD4T	EXV.4	CD4T_CM_$CXCR5^+CXCR3^+$	-0.00922	0.52273	0.02885		N/A
EXV	CD4T	EXV.12	T_H1	-0.00293	0.85909	0.02087		N/A
EXV	CD4T	EXV.20	T_{reg}_$CTLA4^+$	-0.00154	0.88917	0.55744		N/A
EXV	CD4T	EXV.16	T_H17	0.00011	0.89956	0.54946		N/A
EXV	CD4T	EXV.18	T_{reg}_$CD45RA^+CTLA4^+KLRG1^+CXCR3^+$	0.00082	0.4688	0.54946		N/A
EXV	CD4T	EXV.11	CD4T_Naive_$PD1^+ICOS^+$	0.00086	0.31757	0.31161		N/A
EXV	CD4T	EXV.10	CD4T_Naive_$CTLA4^+$	0.001	0.67091	0.15392		N/A
EXV	CD4T	EXV.19	T_{reg}_$CD45RA^+HLADR^+CD38^+CCR6^+CXCR5^+$	0.00112	0.62083	0.90187		N/A
EXV	CD4T	EXV.9	CD4T_Naive_$CD56^+CD16^+CTLA4^+$	0.00309	0.19423	0.76654		N/A

EXV	CD4T	EXV.17	T_{reg}	0.03759	2,00E-05	0.04901		N/A
EXV	CD4T	EXV.8	CD4T_Naive_CD38^{+}	0.04876	0.00795	0.44043	***	N/A
EXV	CD4T	EXV.7	CD4T_Naive	0.06366	1,00E-04	0.01316		N/A
PMA	CD4T	PMA.18	CD4T_CD161^{+}IFNγ^{+}TNF^{hi}	-0.0461	0.000548	0.985486	****	positive
PMA	CD4T	PMA.17	CD4T_CD161^{+}CD27+TNF^{hi}	-0.0438	4,00E-06	0.452442	****	positive
PMA	CD4T	PMA.7	CD4T_CD27^{+}TNF^{hi}	-0.0391	0.026224	0.752944	**	positive
PMA	CD4T	PMA.19	CD4T_IFNγ^{+}TNF^{hi}	-0.0389	0.002432	0.361876	***	positive
PMA	CD4T	PMA.5	CD4T_CD27^{+}IFNγ^{+}TNF^{hi}	-0.0367	0.010022	0.421503	**	positive
PMA	CD4T	PMA.15	CD4T_CD161^{+}CD25^{+}TNF^{+}IL4IL5IL13^{+}	-0.0192	0.000653	0.174203	****	positive
PMA	CD4T	PMA.3	CD4T_CD25^{+}TNF^{+}IL4IL5IL13^{+}	-0.0155	0.171051	0.986917		positive
PMA	CD4T	PMA.16	CD4T_CD161^{+}CD27^{+}	-0.0134	0.193098	0.659724		negative
PMA	CD4T	PMA.14	CD4T_CD161^{+}	-0.0121	0.09213	0.00146	*	negative
PMA	CD4T	PMA.6	CD4T_CD27^{+}PD1^{+}	-0.0078	0.55287	0.386915		negative
PMA	CD4T	PMA.1	CD4T	-0.0048	0.828659	0.174854		negative
PMA	CD4T	PMA.12	CD4T_CD38^{+}CD27^{+}TNF^{+}	-0.002	0.903709	0.048508		positive
PMA	CD4T	PMA.10	CD4T_CD38^{+}CD27^{+}IFNα^{+}IL10^{+}IL4IL5IL13^{+}	0	0.9936	0.928938		positive
PMA	CD4T	PMA.11	CD4T_CD38^{+}CD27^{+}IL10^{+}	0.0013	0.501871	0.94023		positive
PMA	CD4T	PMA.8	CD4T_CD38^{+}CD27^{+}	0.0097	0.708282	0.763083		negative
PMA	CD4T	PMA.4	CD4T_CD27^{+}	0.0134	0.254571	0.236196		negative
PMA	CD4T	PMA.2	CD4T_CD25^{+}CD27^{+}	0.028	1,00E-06	0.443375	*	negative

PMA	CD4T	PMA.13	CD4T_CD38^{hi}CD27^{+}	0.0316	0.042157	0.415696	*	negative
PMA	CD4T	PMA.9	CD4T_CD38^{+}CD27^{+}HLAE^{+}	0.0427	0.058436	0.006181	*	negative

Rows of linear_trend_fdr highlighted in green indicate statistically significant results. *FDR<0.1, **<0.05, ***<0.01, ****<0.001.

Table S9. Differential abundance analysis statistics for Monocyte clusters.

dataset	lineage	cluster	annotation	linear _trend_coef	linear _trend_fdr	quadratic _trend_fdr	significant _linear	cytokine_ expressing
EXV	Myeloids	EXV.1	Monocytes_Classical	-0.024004	0.272022	0.829636		N/A
EXV	Myeloids	EXV.2	Monocytes_Classical_CCR6^{+}	-0.015404	0.309021	0.510096		N/A
EXV	Myeloids	EXV.3	Monocytes_Classical_CD56^{+}	-0.01329	0.003637	0.440433	***	N/A
EXV	Myeloids	EXV.8	Monocytes_NonClassical_KLRG1^{+}	-0.003688	0.796347	0.45979		N/A
EXV	Myeloids	EXV.6	Monocytes_Classical_PD1^{+}ICOS^{+}	7,00E-06	1.0	0.600429		N/A
EXV	Myeloids	EXV.5	Monocytes_Classical_cKit^{+}	0.000598	0.691755	0.588157		N/A
EXV	Myeloids	EXV.7	Monocytes_Intermediate_CD122^{+}Tbet^{+}	0.000612	0.673592	0.956983		N/A
EXV	Myeloids	EXV.4	Monocytes_Classical_CD163^{+}	0.087939	0.001877	0.342457	***	N/A
PMA	Monocytes	PMA.8	Monocytes_TNF^{+}IL1β^{+}	-0.190781	1,00E-06	0.119	***	positive
PMA	Monocytes	PMA.4	Monocytes_CD38^{hi}PDL2^{+}	-0.004852	0.896054	0.952674		positive
PMA	Monocytes	PMA.2	Monocytes_CD16^{+}CD123^{hi}CD38^{hi}PDL2^{+} HLAE^{+}PDL1^{+}	0.004269	0.649337	0.838938		positive
PMA	Monocytes	PMA.7	Monocytes_PDL2^{+}IL1b^{+}	0.007566	0.856457	0.178664		positive
PMA	Monocytes	PMA.6	Monocytes_IL10^{+}IFNα^{+}IL1β^{+}	0.018134	1,00E-06	0.84988	***	positive
PMA	Monocytes	PMA.5	Monocytes_CD123^{hi}CD38^{hi}	0.024193	0.17803	0.001363		negative
PMA	Monocytes	PMA.1	Monocytes	0.046432	0.09213	0.601595		negative
PMA	Monocytes	PMA.3	Monocytes_CD38^{hi}	0.090971	0.002325	0.00158		negative
MPL	Monocytes	MPL1	Monocytes_CD38^{+}CD14^{+}TNF^{+}IL1β^{+}	-0.066182	0.011863	0.756154	***	positive

MPL	Monocytes	MPL5	Monocytes_CD14⁺TNF⁺IL1β⁺	-0.043351	0.00287	0.056974		positive
MPL	Monocytes	MPL4	Monocytes_CD16⁺CD38⁺CD56⁺CD14⁺PDL2⁺HLAE⁺PDL1⁺TNF⁺IL1β⁺	-0.006257	0.705268	0.060855		positive
MPL	Monocytes	MPL2	Monocytes_CD38⁺CD14⁺TNF⁺IL10⁺IL1β⁺	0.022967	0.007255	0.005171		positive
MPL	Monocytes	MPL3	Monocytes_CD38⁺CD14⁺IL1β⁺	0.091948	0.000389	0.236008	***	positive

Rows of linear_trend_fdr highlighted in green indicate statistically significant results. *FDR<0.1, **<0.05, ***<0.01, ****<0.001.

Table S10. List of IgG1 and IgG2 analytes.

Number	IgG1	IgG2
1	H3N4	H3N4
2	H3N5	H4N4
3	H4N4	H5N4
4	H4N5	H3N3F1
5	H5N4	H3N4F1
6	H3N3F1	H3N5F1
7	H3N4F1	H4N3F1
8	H3N5F1	H4N4F1
9	H4N3F1	H4N5F1
10	H4N4F1	H5N4F1
11	H4N5F1	H5N5F1
12	H5N4F1	H6N4F1
13	H5N5F1	H4N4F2
14	H6N4F1	H5N4F2
15	H4N4S1	H4N3F1S1
16	H5N4S1	H4N4F1S1
17	H4N3F1S1	H4N5F1S1
18	H4N4F1S1	H5N3F1S1
19	H4N5F1S1	H5N4F1S1
20	H5N4F1S1	H5N5F1S1
21	H5N4F2S1	H6N3F1S1
22	H5N5F1S1	H5N4F1S2
23	H6N3F1S1	
24	H5N4F1S2	

The table specifies for each IgG subclass the analytes that were quantified and used for calculating derived traits in terms of glycan composition HwNxFySz. Monosaccharides are indicated by G (galactose), F (fucose), N (N-acetyl glucosamine), and S (sialic acid).

Table S11. Mass cytometry panel 1: ex vivo phenotyping.

Tag	Target	Dilution	Company	Cat#	Clone
^{89}Y	CD45	200	Fluidigm	3089003B	HI30
^{102}Pd	20-Plex Pd Barcoding Kit		Fluidigm	201060	
^{104}Pd	20-Plex Pd Barcoding Kit		Fluidigm	201060	
^{106}Pd	20-Plex Pd Barcoding Kit		Fluidigm	201060	
^{108}Pd	20-Plex Pd Barcoding Kit		Fluidigm	201060	
^{110}Pd	20-Plex Pd Barcoding Kit		Fluidigm	201060	
^{115}In	CD57	50	Biolegend	393302	QA17A04
^{141}Pr	CCR6 (CD196)	50	Biolegend	353427	G034E3
^{142}Nd	CD19	200	Biolegend	302202	HIB19
^{143}Nd	CD117 (cKIT)	50	Biolegend	313223	104D2
^{145}Nd	CD4	200	Biolegend	300541	RPA-T4
^{146}Nd	CD8a	200	Biolegend	100755	53-6.7
^{147}Sm	CXCR3	400	Biolegend	353733	G025H7
^{148}Nd	CD14	100	Biolegend	301843	M5E2
^{149}Sm	CD25	50	Biolegend	101913	3C7
^{150}Nd	CXCR5	50	Biolegend	356902	J252D4
^{151}Eu	CD123	50	Biolegend	306027	6H6
^{152}Sm	TCRgd	50	Biolegend	331202	B1
^{153}Eu	CD7	400	Biolegend	343111	CD7-6B7
^{154}Sm	CD163	50	Biolegend	333602	GHI/61
^{155}Gd	CD127 (IL-7Ra)	100	Biolegend	351337	A019D5
^{156}Gd	CRTh2 (CD294)	50	Biolegend	350102	BM16
^{158}Gd	CD122 (IL-2Rb)	50	Biolegend	339015	TU27
^{159}Tb	CD197 (CCR7)	50	Biolegend	353237	G043H7
^{160}Gd	FOXP3	50	Biolegend	320202	259D
^{161}Dy	KLRG1	100			
^{162}Dy	CD11c	200	Biolegend	337221	Bu15
^{163}Dy	CD152 (CTLA4)	50	Biolegend	369602	BNI3
^{164}Dy	CD161	200	Biolegend	339919	HP-3G10
^{165}Ho	CD278 (ICOS)	50	Miltenyi	130-122-304	REA192
^{166}Er	Tbet	50	Biolegend	644825	4B10
^{167}Er	CD27	100	Biolegend	302839	O323
^{168}Er	HLA-DR	400	Biolegend	307651	L243
^{169}Tm	GATA3	50	Miltenyi	130-108-061	REA174
^{170}Er	CD3	200	Biolegend	300443	UCHT1
^{171}Yb	RORgt	50	Biolegend	654302	2F7-2
^{172}Yb	CD38	200	Biolegend	303535	HIT2
^{173}Yb	CD45RO	400	Biolegend	304239	UCHL1
^{174}Yb	NKp46 (CD335)	200	Biolegend	331902	9.00E+02
^{175}Lu	PD-1	200	Biolegend	329941	EH12.2H7
^{176}Yb	CD56	200	Biolegend	318345	HCD56
$^{191/193}$Ir	DNA-Intercalator		Fluidigm	201192A	
^{194}Pt	Viability		Fluidigm	201194	
^{198}Pt	CD45RA	100	Biolegend	304143	HI100
^{209}BI	CD16	50	Biolegend	302051	3G8

Table S12. Mass cytometry panel 2: cytokine production.

Tag	Marker	Dilution	Company	Cat#	Clone
**^{89}Y**	CD45	200	Fluidigm	3089003B	HI30
**^{102}Pd**	20-Plex Pd Barcoding Kit		Fluidigm	201060	
**^{104}Pd**	20-Plex Pd Barcoding Kit		Fluidigm	201060	
**^{106}Pd**	20-Plex Pd Barcoding Kit		Fluidigm	201060	
**^{108}Pd**	20-Plex Pd Barcoding Kit		Fluidigm	201060	
**^{110}Pd**	20-Plex Pd Barcoding Kit		Fluidigm	201060	
**^{115}In**	CD57	50	Biolegend	393302	QA17A04
**^{142}Nd**	CD19	200	Biolegend	302202	HIB19
**^{144}Nd**	PD-L2	50	Biolegend	329613	24F.10C12
**^{145}Nd**	CD4	200	Biolegend	300541	RPA-T4
**^{146}Nd**	CD8	200	Biolegend	100755	53-6.7
**^{147}Sm**	HLA-E	50	Biolegend	342602	3D12
**^{148}Nd**	CD14	100	Biolegend	301843	M5E2
**^{149}Sm**	CD25	50	Biolegend	101913	3C7
**^{151}Eu**	CD123	50	Biolegend	306027	6H6
**^{152}Sm**	TCRγδ	50	Biolegend	331202	B1
**^{153}Eu**	Tim3 (CD366)	50	Biolegend	345019	F38-2E2
**^{154}Sm**	CD163	50	Biolegend	333602	GHI/61
**^{155}Gd**	IFNγ	400	Biolegend	506521	B27
**^{159}Tb**	IL-1β	50	Thermo Fisher	14-7018-81	CRM56
**^{160}Gd**	TNF	400	Biolegend	502941	MAb11
**^{162}Dy**	CD11c	200	Biolegend	337221	Bu15
**^{164}Dy**	CD161	200	Biolegend	339919	HP-3G10
**^{165}Ho**	IFNα	50			
**^{166}Er**	IL-10	50	Biolegend	501423	JES3-9D7
**^{167}Er**	CD27	100	Biolegend	302839	O323
**^{168}Er**	HLA-DR	400	Biolegend	307651	L243
**^{169}Tm**	IL-4	200	Biolegend	500829	MP4-25D2
**^{169}Tm**	IL-5	200	Biolegend	504309	TRFK5
**^{169}Tm**	IL-13	200	Biolegend	501901	JES10-5A2
**^{170}Er**	CD3	200	Biolegend	300443	UCHT1
**^{172}Yb**	CD38	200	Biolegend	303535	HIT2
**^{173}Yb**	PD-L1 (CD274)	100	Biolegend	329719	29E.2A3
**^{174}Yb**	NKp46	200	Biolegend	331902	9.00E+02
**^{175}Lu**	PD-1	200	Biolegend	329941	EH12.2H7
**^{176}Yb**	CD56	50	Biolegend	318345	HCD56
**$^{191/193}$Ir**	DNA-Intercalator		Fluidigm	201192A	
**^{194}Pt**	Viability		Fluidigm	201194	
**^{209}BI**	CD16	50	Biolegend	302051	3G8

Table S13. Spectral flow cytometry antibody panel 1.

Fluorophore	Target	Clone	Company	Cat#	Dilution
**Zombie NIR**	Live/dead	N/A	BioLegend	423105	5000
**AF700**	CD45	2D1	Biolegend	368513	100
**CF568**	CD45	2D1	Biolegend	368502	100
**PB**	CD45	2D1	Biolegend	368539	100
**BUV661**	BDCA-2	V24-785	BD	749920	100
**BUV615**	CD11c	3.9 (RUO)	BD	752323	50
**BV570**	CD14	M5E2	Biolegend	301831-32	100
**BV750**	CD141	1A4	BD	747244	200
**BUV563**	CD16	3G8	BD	748851	800
**BV605**	CD161	HP-3G10	BioLegend	339916	100
**BUV395**	CD19	HIB10	BioLegend	740287	50
**BUV805**	CD1c	F10/21A3	ThermoFisher	748722	200
**BUV496**	CD3	UCHT1	BD	612940	50
**SB550**	CD4	SK3	Biolegend	344656	50
**BV650**	CD45RO	UCHL1	BioLegend	304232	50
**BUV737**	CD56	NCAM16.2	BD	612767	200
**BV510**	CD8	RPA-T8	Biolegend	301047-8	100
**BV785**	CRTH2	BM16	BioLegend	350124	50
**APCFire810**	HLA-DR	L243	Biolegend	307673	100
**Percpe710**	TCRyd	REA591	Miltenyi Biotec	130-114-040	50
**PE-Fire810**	CCR7	G043H7	BioLegend	353269	50
**BV785**	Tbet	4B10	BioLegend	644835	100
**PE/Dazzle**	Foxp3	206D	BioLegend	320126	50
**PE**	PKM	EPR10138B	Abcam	ab206129	200
**AF488**	ACC1	EPR23235-147	Abcam	ab272704	200
**DL405**	GLUT1	EPR3915	Abcam	ab252403	200
**AF647**	SDHA	EPR9043B	Abcam	ab240098	200
**APC/Cy7**	G6PD	EPR20668	Abcam	ab231828	200
**PE/Cy7**	Cytc	7H8.2C12	Abcam	ab237966	200
**PE/Cy5**	CPT1A	EPR21843-71-2F	Abcam	ab235841	200

Table S14. Spectral flow cytometry antibody panel 2.

Fluorophore	Target	Clone	Supplier	Cat #	Dilution
Zombie NIR	Dead		Biolegend	423106	500
BUV563	CD16	3G8	BD Bioscience	148851	1600
BV510	CD57	QA17A04	BioLegend	393314	1600
BYG750	CD4	SK3	Cytek	R7-20160	800
SB550	CD8	SK1	Biolegend	344759	800
AF700	HLADR	L243	Biolegend	307626	800
PECF594	CD11c	B-ly6	BD Bioscience	562393	800
v450	CD45RA	HI100	BD Bioscience	560362	200
BUV496	CD3	UCHT1	BD Bioscience	612940	100
BUV737	CD56	NCAM16.2	BD Bioscience	612766	100
Vio700	TCRgd	RA591	Miltenyi Biotec	130-113-514	100
APC/Fire810	CD38	HIT2	BioLegend	303549	100
BV570	CD14	M5E2	BioLegend	301832	100
Spark685NIR	CD19	HIB19	BioLegend	302269	100
PE/Fire810	CD197	G043H7	BioLegend	353269	50
BUV805	CD161	HP-3G10	BD Bioscience	749221	50
BV605	NKG2C	134591	BD Bioscience	748166	50
SB645	CD27	O323	Thermo Fisher	64-0279-42	50
FITC	TNF	W19063E	BioLegend	376208	1600
APC	IFNγ	4S.B3	BioLegend	502512	200
